# Endosperm cellularization failure induces a dehydration-stress response leading to embryo arrest

**DOI:** 10.1093/plcell/koac337

**Published:** 2022-11-25

**Authors:** Wenjia Xu, Hikaru Sato, Heinrich Bente, Juan Santos-González, Claudia Köhler

**Affiliations:** Swedish University of Agricultural Sciences & Linnean Center for Plant Biology, Uppsala BioCenter, Almas Allé 5, SE-750 07 Uppsala, Sweden; Swedish University of Agricultural Sciences & Linnean Center for Plant Biology, Uppsala BioCenter, Almas Allé 5, SE-750 07 Uppsala, Sweden; Swedish University of Agricultural Sciences & Linnean Center for Plant Biology, Uppsala BioCenter, Almas Allé 5, SE-750 07 Uppsala, Sweden; Max Planck Institute of Molecular Plant Physiology, Am Mühlenberg 1, 14476 Potsdam-Golm, Germany; Swedish University of Agricultural Sciences & Linnean Center for Plant Biology, Uppsala BioCenter, Almas Allé 5, SE-750 07 Uppsala, Sweden; Swedish University of Agricultural Sciences & Linnean Center for Plant Biology, Uppsala BioCenter, Almas Allé 5, SE-750 07 Uppsala, Sweden; Max Planck Institute of Molecular Plant Physiology, Am Mühlenberg 1, 14476 Potsdam-Golm, Germany

## Abstract

The endosperm is a nutritive tissue supporting embryo growth in flowering plants. Most commonly, the endosperm initially develops as a coenocyte (multinucleate cell) and then cellularizes. This process of cellularization is frequently disrupted in hybrid seeds generated by crosses between different flowering plant species or plants that differ in ploidy, resulting in embryo arrest and seed lethality. The reason for embryo arrest upon cellularization failure remains unclear. In this study, we show that triploid *Arabidopsis thaliana* embryos surrounded by uncellularized endosperm mount an osmotic stress response that is connected to increased levels of abscisic acid (ABA) and enhanced ABA responses. Impairing ABA biosynthesis and signaling aggravated triploid seed abortion, while increasing endogenous ABA levels as well as the exogenous application of ABA-induced endosperm cellularization and suppressed embryo growth arrest. Taking these results together, we propose that endosperm cellularization is required to establish dehydration tolerance in the developing embryo, ensuring its survival during seed maturation.

IN A NUTSHELL

**Background:** In most angiosperms, the endosperm initiates as a coenocyte and starts to cellularize after a defined number of nuclear divisions. This process of cellularization is frequently disrupted in hybrid seeds generated after crosses between different flowering plant species or plants that differ in ploidy and is thus a major obstacle to plant breeding. Restored endosperm cellularization allows researchers to rescue hybrid embryos, revealing an essential role of this process for embryo survival. Despite the importance of endosperm cellularization, why this developmental transition causes embryo arrest remained unknown.

**Question:** To address the functional importance of endosperm cellularization for embryo survival, we generated transcriptome data of triploid embryos that are surrounded by uncellularized endosperm and triploid embryos surrounded by cellularized endosperm.

**Findings:** Here we showed that embryos surrounded by an uncellularized endosperm mount an osmotic stress response that is connected to increased levels of ABA and ABA responses. By manipulating ABA biosynthesis and signaling, we revealed a causal connection between ABA-mediated osmotic stress and embryo arrest. Based on these data we propose that endosperm cellularization is required to establish dehydration tolerance in the developing embryo, ensuring survival during seed maturation. Researchers have known for decades that endosperm cellularization is essential for embryo survival, but what makes this transition relevant remained unknown. Our work provides important insights into this phenomenon.

**Next steps:** We found that ABA can suppress triploid seed abortion, but the detailed mechanism remains to be explored. We would like to understand the role of the ABA pathway for embryo survival by characterizing when, where, and how it acts during seed development.

## Introduction

The endosperm is a developmental innovation of flowering plants supporting embryo growth and germination. This tissue is a product of the double fertilization process, whereby one sperm cell fertilizes the haploid egg cell, giving rise to the diploid embryo, and the other sperm cell fertilizes the predominantly diploid central cell, giving rise to the triploid endosperm (Costa et al., 2012). In most flowering plants, the endosperm initially undergoes multiple rounds of nuclear divisions that are not followed by cellularization, giving rise to a multinucleate cell known as a coenocyte. Cellularization is initiated after a defined number of nuclear divisions and ultimately fills the complete chamber of the central cell (Costa et al., 2012). The timing of endosperm cellularization is important for embryo survival and is a critical determinant of final seed growth (Hehenberger et al., 2012; Batista et al., 2019).

In *Arabidopsis thaliana*, endosperm cellularization starts when the embryo has reached the heart stage of development at approximately 4 days after pollination (DAP) and is completed when the embryo has reached the torpedo stage at ∼6 DAP (Boisnard-Lorig et al., 2001). Crosses between different flowering plant species or plants that differ in ploidy (interspecies and interploidy hybridizations, respectively) frequently result in the failure of endosperm cellularization and embryo arrest for reasons that remain to be defined (Ramsey and Schemske, 1998; Lafon-Placette and Kohler, 2016; Köhler et al., 2021). In Arabidopsis, crosses between diploid maternal plants and tetraploid pollen donors give rise to triploid seeds containing embryos that arrest at the torpedo stage of seed development surrounded by a largely uncellularized endosperm. The resulting seeds collapse and fail to germinate, a phenomenon that has been termed the “triploid block” (Scott et al., 1998).

Embryos from interploidy and interspecies hybridizations over a wide range of species can be rescued by removing them from the seed and incubating them in vitro on suitable media, supporting the causal role of the endosperm in embryo arrest (Fratini and Ruiz, 2011; Roy et al., 2011; Ji et al., 2015; Rebernig et al., 2015). Furthermore, genetic repressors of the triploid block that have been identified in Arabidopsis restore endosperm cellularization, emphasizing the finding that endosperm cellularization is essential for embryo survival (Kradolfer et al., 2013; Wolff et al., 2015; Jiang et al., 2017). One of the identified suppressors of the triploid block is a mutation of *NRPD1* (*NUCLEAR RNA POLYMERASE D1*), encoding the largest component of RNA polymerase IV (Pol IV; Erdmann et al., 2017; Martinez et al., 2018). Pol IV generates precursor RNAs that are converted into 24-nt short-interfering RNAs, which initiate RNA-directed DNA methylation (RdDM; Rymen et al., 2020). Similar to many other suppressors of the triploid block, paternal inheritance of the *nrpd1* mutation is sufficient to suppress the triploid block in Arabidopsis (Erdmann et al., 2017; Martinez et al., 2018).

In this study, we aimed to decipher the cause of embryo arrest upon endosperm cellularization failure. To this end, we performed transcriptome analyses of triploid torpedo-stage Arabidopsis embryos isolated from seeds containing cellularized and uncellularized endosperm. We found that embryos surrounded by an uncellularized endosperm elicited an osmotic stress response that was connected with increased abscisic acid (ABA) levels and responses. Impaired ABA synthesis and signaling aggravated the triploid block, while increasing endogenous ABA levels or the exogenous application of ABA-induced endosperm cellularization and suppressed the triploid block, revealing a role for ABA in endosperm-mediated embryo arrest. These findings suggest that endosperm cellularization is required to confer dehydration tolerance to the developing embryo, ensuring its survival during seed maturation.

## Results

### Endosperm cellularization failure causes the expression of osmotic stress-response genes in triploid embryos

To decipher the molecular mechanism causing embryo arrest upon failure of endosperm cellularization, we compared the transcriptome profiles of isolated triploid embryos surrounded by uncellularized *versus* cellularized endosperm. Crosses of diploid wild-type plants with tetraploid pollen donors (2x wt × 4x wt) give rise to triploid (3x) embryos surrounded by uncellularized endosperm, while crosses of diploid wild-type plants with tetraploid *nrpd1* pollen donors (2x wt × 4x *nrpd1*) give rise to 3x embryos mostly surrounded by cellularized endosperm (Martinez et al., 2018). We also generated transcriptome profiles of diploid wt (2x wt × 2x wt) and diploid *nrpd1* (2x wt × 2x *nrpd1*) embryos, referred to as 2x wt and 2x *nrpd1*, respectively. Consistent with previous data (Martinez et al., 2018), crosses between 2x wt and 4x wt resulted in 3x seed abortion at a frequency of more than 70%, while crosses between 2x wt and 4x *nrpd1* strongly suppressed seed abortion to a frequency of 8% (Supplemental Figure S1). The isolated embryos were at the torpedo stage of development, which corresponded to 6 DAP for 3x *nrpd1* embryos but 8 DAP for 3x wt embryos (Supplemental Figure S1, B–E). Thus, embryo growth of 3x *nrpd1* was not arrested but was still delayed compared to 2x wt or 2x *nrpd1* embryos. Transcriptome profiles were generated in biological triplicates using separate crosses from distinct plants (Supplemental Table S1).

**Figure 1:**
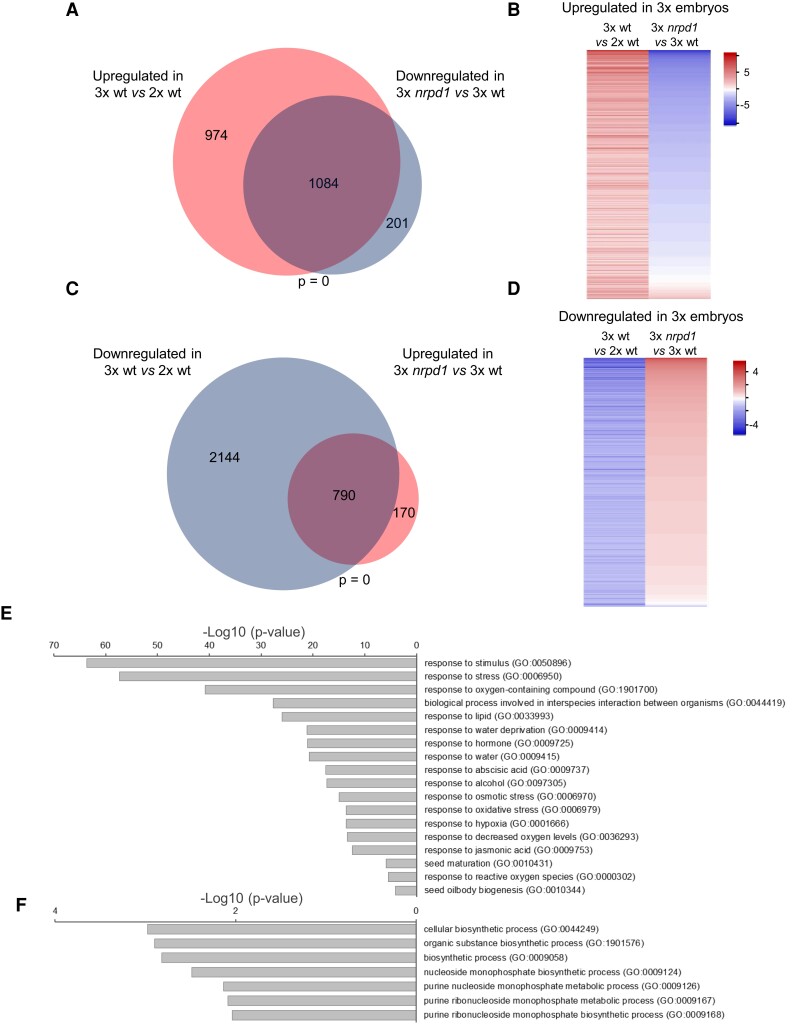
Failure of endosperm cellularization induces stress-response genes in 3x embryos. A, C, Venn diagrams show the overlap of genes that are significantly up-regulated in 3x wt embryos versus 2x wt embryos and down-regulated in 3x *nrpd1* embryos versus 3x wt embryos (A) vice versa for the overlap of genes that are significantly down-regulated 3x wt embryos versus 2x wt embryos and up-regulated in 3x *nrpd1* embryos versus 3x wt embryos (C). Significance of the overlap was calculated using a hypergeometric test. B, D, Heat map of log2 fold expression changes of genes that were either twofold up-regulated (B) or down-regulated (D) in 3x wt embryos versus 2x wt embryos and their corresponding expression in 3x *nrpd1* embryos versus 3x wt embryos. E, Significantly enriched GO terms for overlapping genes shown in (A; 1,084 genes, adjusted *P*-value <0.0001). F, Significantly enriched GO terms for overlapping genes shown in (C; 790 genes, adjusted *P*-value <0.01). GO enrichment was analyzed on http://geneontology.org. The complete list of GO terms is provided in the Supplemental Data.

Based on tissue-specific transcript analysis (Schon and Nodine, 2017), the samples mainly contained embryo transcripts with negligible endosperm contamination (Supplemental Figure S2). We identified 2,058 up-regulated genes [log2(fold change) > 1, *P* < 0.05] in 3x wt compared to 2x wt embryos, approximately half (53%) of which became down-regulated in 3x *nrpd1* embryos [log2 (fold change) < −1, *P* < 0.05; Figure 1, A and B, Supplemental Data Set 1]. Similarly, out of 2,934 down-regulated genes in 3x wt embryos, a significant number (790, *P* = 0, hypergeometric test) became up-regulated in 3x *nrpd1* embryos (Figure 1, C and D). These data indicate that restored endosperm cellularization restores normal embryo development. Consistently, in contrast to a large number of differentially expressed genes (DEGs) between 3x and 2x wt embryos, there were only 382 DEGs between 3x and 2x *nrpd1* embryos. We identified only 9 DEGs in 2x *nrpd1* compared to 2x wt embryos, revealing that the mutation of *NRPD1* had only minor effects on 2x embryo development (Supplemental Table S2).

Gene ontology (GO) enrichment analysis of up-regulated genes in 3x wt versus 2x wt revealed that these genes were strongly enriched for GO terms related to ABA metabolism and responses, as well as responses to oxidative and osmotic stress, seed maturation, and dormancy (Figure 1E). Conversely, down-regulated genes were enriched for GO terms related to cellular biosynthetic processes and purine metabolic processes (Figure 1F). Supplemental Data Set 2 provides a full list of GO terms. Based on these data, we hypothesized that endosperm cellularization failure causes a stress response, resulting in growth arrest of the embryo.

### Endosperm cellularization failure induces an embryo-specific seed maturation response

**Figure 2:**
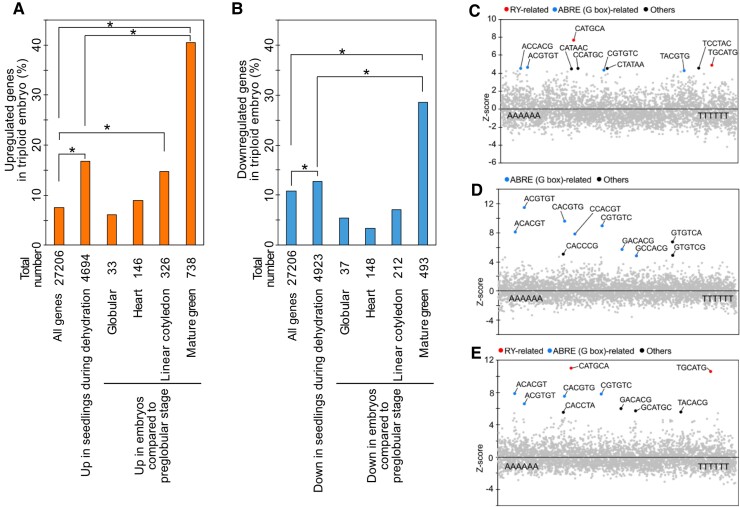
RY motifs and ABRE-related elements are enriched among 1 kb-promoter regions of up-regulated genes in 3x embryos. A, B, Plots show the percentage of up- and down-regulated genes in 3x embryos versus 2x embryos. Seed maturation-inducible genes in developing embryos were compared with dehydration-inducible genes in seedlings. Asterisks indicate significant differences (*P* < 0.0005, pairwise Fisher's exact test). C–E, Overrepresentation analysis of hexamer motifs in the 1-kb promoter regions of the top 100 up-regulated genes in 3x embryos versus 2x embryos compared to those of all Arabidopsis genes (C), in the 1-kb promoters of the top 100 up-regulated genes in seedlings during dehydration stress versus control conditions compared to those of all Arabidopsis genes (D) and in the 1-kb promoters of the top 100 up-regulated genes in green mature embryos versus preglobular embryos compared to those of all *Arabidopsis* genes (E). *Z* scores (*y* axis) for the observed frequencies of all hexamer motifs (*x* axis) are presented in the scatter plot. The top 10 enriched motifs among each set of genes are highlighted.

To test the hypothesis that endosperm cellularization failure initiates a stress response, we compared the identified DEGs in 3x embryos with previously published transcriptome data of seedlings under dehydration-stress conditions (Sato et al., 2018). The results revealed that up-regulated and down-regulated genes in 3x embryos and seedlings during dehydration stress had significant overlaps (Figure 2, A and B). We compared the frequency of hexamer sequences within the 1 kb upstream sequences of the top 100 up-regulated genes in 3x embryos with their normalized frequencies in the promoters of genes in the entire Arabidopsis genome. We found that RY motif (CATGCATG)-related sequences were highly enriched in the upstream regions of up-regulated genes (Figure 2C), in contrast to the reported enrichment of ABA-responsive elements (ABREs) [(C/T)ACGTGGC] among up-regulated genes in seedlings during dehydration stress (Maruyama et al., 2012). We confirmed that ABREs, but not RY motifs, were enriched using the dehydration-stress-responsive transcriptome data (Figure 2D). These data suggest that osmotic stress responses in 3x embryos are regulated by different factors from those in seedlings.

Seed maturation provides desiccation tolerance to mature seeds, and transcriptome changes during seed maturation were reported to be similar to those during drought stress responses in seedlings (Nakashima et al., 2014; Chauffour et al., 2019). In particular, B3-type transcription factors such as ABI3 (ABA INSENSITIVE 3) and FUS3 (FUSCA3), which target RY motifs, play critical roles in the process of seed maturation (Vicente-Carbajosa and Carbonero, 2005; Wang and Perry, 2013; Tian et al., 2020). Comparing the DEGs in 3x embryos with both dehydration-stress-responsive genes in seedlings (Sato et al., 2018) and inducible genes during seed maturation in embryos (Belmonte et al., 2013), we found that the DEGs in 3x embryos were more highly associated with seed maturation than dehydration-stress responses in seedlings (Figure 2, A and B). Additionally, consistent with previous studies, we confirmed that RY motifs were most highly enriched among inducible genes during seed maturation in embryos (Figure 2E), and the enriched hexamers among the promoters of up-regulated genes in 3x embryos had higher similarity to those among inducible genes during seed maturation (Figure 2C). These data suggest that the triploid block induces an embryo-specific seed maturation response that resembles a dehydration response, which is mainly regulated by B3-type transcription factors.

To further test the similarity of dehydration-specific gene expression with the transcriptome changes in 3x embryos, we divided the DEGs during dehydration stress (Sato et al., 2014) and seed maturation (Belmonte et al., 2013) into three groups: (1) dehydration-stress-specific genes, (2) seed maturation-specific genes, and (3) common genes between dehydration and seed maturation (Supplemental Figure S3, A and B). Every group significantly overlapped with the DEGs in 3x wt embryos relative to 2x wt embryos (Supplemental Figure S3, C and D), revealing that the seed maturation response mounted in 3x wt embryos shares similarities with dehydration-stress responses. This finding is consistent with the fact that seed maturation induces cellular dehydration (Finkelstein et al., 2002).

### ABA levels and response are enhanced in triploid embryos

**Figure 3:**
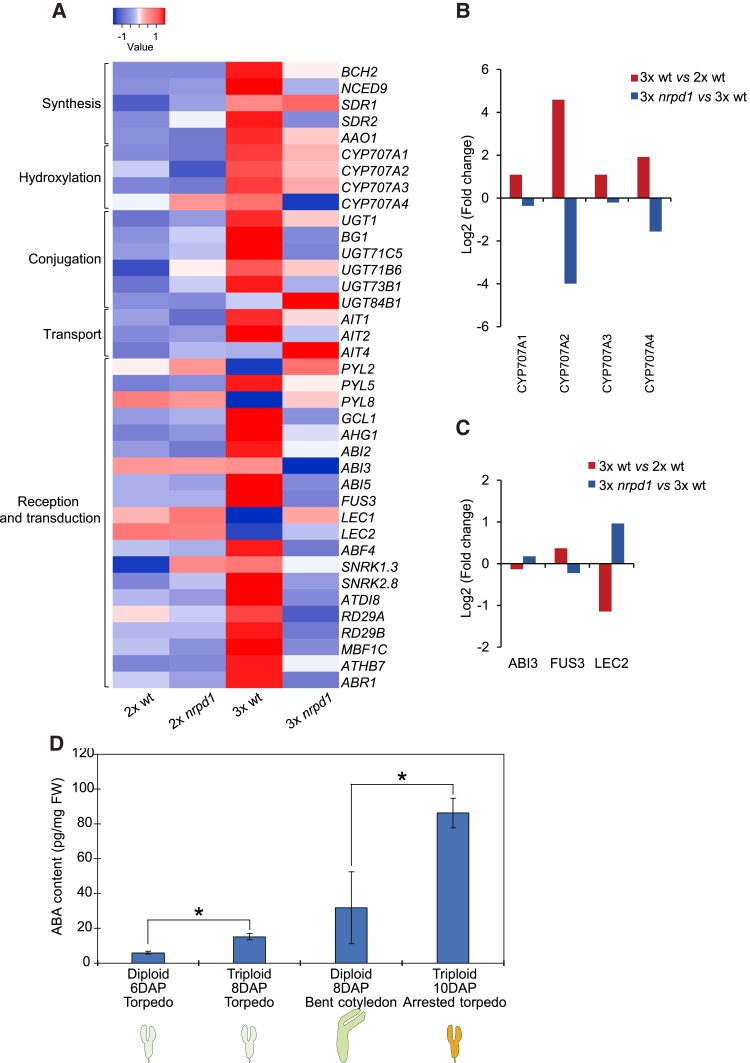
ABA metabolism and signaling are highly activated in 3x embryos. A, Heat map showing the expression profiles of ABA-related genes in 2x and 3x embryos surrounded by cellularized (2x wt, 2x *nrpd1*, 3x *nrpd1*) and uncellularized endosperm (3x wt). Each row represents a gene, and each column represents a sample type. The gene expression levels are represented as log_10_TPM (transcript per million reads); low values (−1 to 0) and high values (0 to 1) are shown. B, C, Bar graphs of log2 fold expression changes in 3x wt embryos versus 2x wt embryos (upward bars) and 3x *nrpd1* embryos versus 3x wt embryos (downward bars) of genes for ABA hydroxylation (B) and B3 type transcription factors that induce seed maturation (C). D, ABA levels are elevated in triploid seeds. Total ABA levels were measured by GC-MS from seeds derived from 2x wt and 3x wt at the torpedo or later stage. An average of four biological replicates (derived from independent crosses of distinct plants) and two technical repeats is shown. Error bars correspond to standard deviation. FW and DAP indicate fresh weight and days after pollination, respectively. Typical developmental stages in each seed are shown below the bars. Asterisks indicate significant differences. *, *P* < 0.01 (Student's *t* test).

ABA is the major phytohormone involved in osmotic stress responses (Nakashima et al., 2014; Yoshida et al., 2014). We analyzed the overlapping DEGs in 3x wt versus 2x wt and 3x wt versus 3x *nrpd1* using the Kyoto Encyclopaedia of Genes and Genomes (KEGG) mapper (Kanehisa and Goto, 2000) for plant hormone signal transduction pathways. Many input DEGs mapped to key regulators of the ABA signal transduction pathway (Supplemental Figure S4A) but not to other hormone transduction pathways. We also identified DEGs that mapped to major nodes of ABA biosynthesis and catabolism (Supplemental Figure S4B), revealing that the ABA biosynthesis and signaling pathway is misregulated in 3x embryos. To identify which genes were specifically affected, we investigated the expression of genes that were previously linked to ABA synthesis, degradation, and signal transduction (Nambara and Marion-Poll, 2005; Finkelstein, 2013). Out of 157 genes, 31 (19.7%) were up-regulated in 3x versus 2x wt and down-regulated in 3x *nrpd1* versus 3x wt embryos (Figure 3A), which is significantly more than expected by chance (7.9e − 13, hypergeometric test).

Interestingly, the misregulated genes included the ABA biosynthesis gene *NCED9* (Figure 3A), which was previously shown to regulate ABA accumulation during seed maturation (Lefebvre et al., 2006). We also found that the SNRK1 kinase-encoding gene *SNRK1.3* was up-regulated in 3x wt embryos (Figure 3A). SNRK1 kinases activate the transcription factor FUS3 via phosphorylation (Tsai and Gazzarrini, 2012), which is required for normal seed development (Chan et al., 2017). These data are in agreement with the enriched FUS3 targeted RY motifs in the upstream regions of up-regulated genes in 3x embryos (Figure 2C). Additionally, we found that the expression level of *FUS3* itself was also slightly but significantly up-regulated in 3x versus 2x wt embryos (Figure 3C), which is consistent with the induction of *SNRK1.3* and overrepresentation of genes with RY motifs (Figure 2C). The up-regulated gene *SNRK2-8* belongs to subclass II of SNRK2 kinases, whose detailed molecular roles in drought stress responses remain unclear (Mizoguchi et al., 2010; Kulik et al., 2011), while subclass III SNRK2s are major regulators of ABA signaling in seedlings (Fujita et al., 2013). Overall, these results indicate that ABA pathway activity is elevated in 3x embryos and is suppressed in 3x *nrpd1* embryos. They also suggest that the ABA response activated in 3x embryos differs from known drought stress responses in seedlings.

To directly test whether ABA levels were indeed altered in 3x embryos, we measured ABA levels in 2x and 3x seeds containing torpedo-stage and bent cotyledon-stage embryos using liquid chromatography-mass spectrometry (LC-MS). Consistent with the transcriptome data, 8 DAP 3x wt seeds containing torpedo-stage embryos had nearly three times higher levels of ABA compared to 6 DAP 2x wt seeds containing embryos at the same developmental stage (Figure 3C). Furthermore, 3x seeds with arrested torpedo-stage embryos and uncellularized endosperm had significantly higher ABA levels than 8 DAP 2x seeds containing bent cotyledon-stage embryos. Thus, at similar stages of embryo development, 3x seeds had significantly higher levels of ABA than 2x seeds, which increased even further upon embryo arrest.

To further investigate cell type-specific ABA accumulation in 2x and 3x wt seeds, we measured ABA levels *in planta* using the ABA reporter ABAleon2.1 (Waadt et al., 2014). This reporter allows relative amounts of ABA to be measured by calculating the emission ratios of two fluorescent proteins, mTurquoise and cpVenus173, whereby increased ratios of mTurquoise to cpVenus173 indicate a higher accumulation of ABA. We analyzed ABA levels in three seed compartments: (1) micropylar regions with embryos, (2) central, and (3) chalazal regions (Supplemental Figure S5). The micropylar and central regions had significantly higher levels of ABA in 3x compared to 2x seeds (Supplemental Figure S5), which is consistent with our finding that 3x wt embryos had increased osmotic stress responses (Figure 1).

### Inhibiting ABA catabolism suppresses triploid seed abortion

The transcriptional responses of 3x embryos to osmotic stress and ABA (Figure 1E) together with their increased ABA levels (Figure 3D) raised the possibility that 3x embryos suffer from osmotic stress. In addition to the increased expression of the ABA biosynthesis gene *NCED9* in 3x embryos, we also observed highly increased expression of the ABA hydroxylase-encoding gene *CYP707A2* (Figure 3, A and B). CYP707A2 belongs to a family of cytochrome P450 monooxygenases that catabolize ABA by converting it to 8-Hydroxy ABA, which spontaneously isomerizes to phaseic acid and is then further reduced to dihydrophaseic acid (DPA; Krochko et al., 1998). CYP707A2 regulates ABA levels from late seed maturation to germination, and mutants of *CYP707A2* have increased levels of ABA (Kushiro et al., 2004; Okamoto et al., 2006). The activation of ABA pathways was previously shown to increase osmotic stress tolerance and survival in plants (Iuchi et al., 2001; Fujita et al., 2005; Cao et al., 2017). Furthermore, ABA was proposed to promote endosperm cellularization (Cheng et al., 2014), raising the question of whether the depletion of CYP707A2 function would suppress triploid seed arrest.

**Figure 4:**
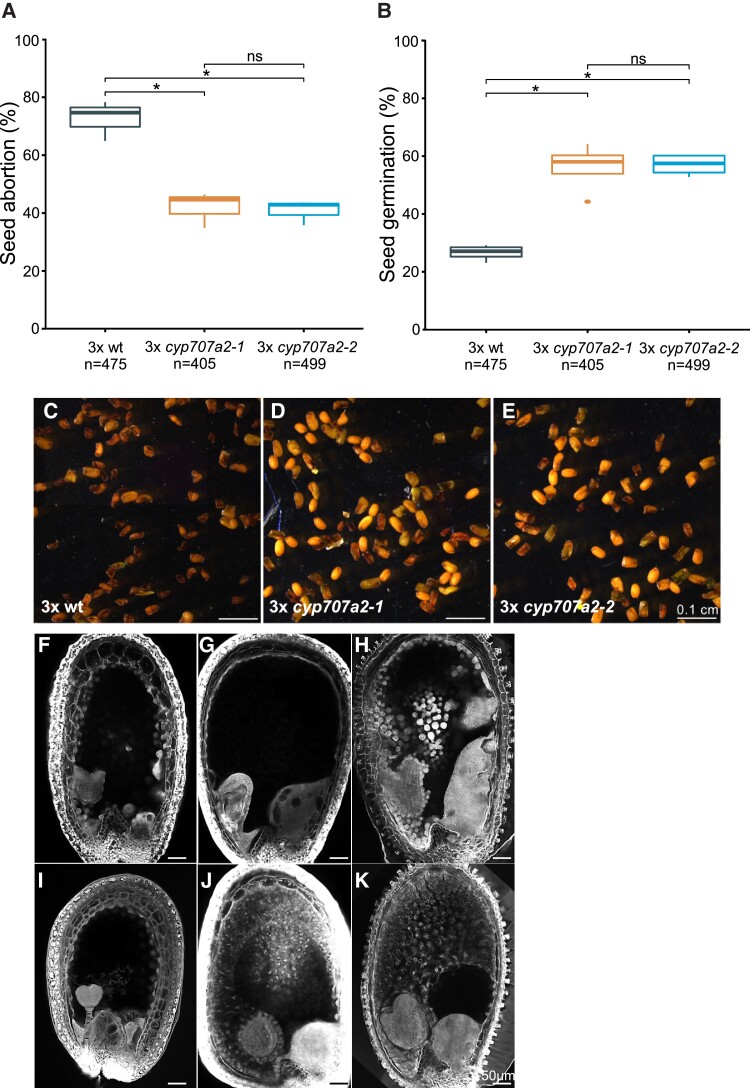
Knockout of *CYP707A2* suppresses triploid seed abortion. A, Phenotypic classification of hybrid seeds 3x wt (2x wt × 4x wt), 3x *cyp707a2-1* (2x *cyp707a2-1* × 4x *cyp707a2-1*), and 3x *cyp707a2-2* (2x *cyp707a2-2* × 4x *cyp707a2-2*) and their corresponding germination frequency (B). Boxes show medians and the interquartile range, and whiskers indicate the full range. Numbers below the plots correspond to the numbers of analyzed seeds. Asterisks indicate significant differences. ns, not significant. *, *P* < 0.01, relative to the control (Tukey's HSD). C–E, Dry seed morphology is shown for 3x wt (C), 3x *cyp707a2-1* (D), and 3x *cyp707a2-2* (E). F–K, Endosperm cellularization as determined by Feulgen staining at 6 DAP for 3x wt seeds (F) and 3x *cyp707a2-1* seeds (I), 8 DAP for 3x wt seeds (G) and 3x *cyp707a2-1* seeds (J), and 9 DAP for 3x wt seeds (H) and 3x *cyp707a2-1* seeds (K). Scale bars correspond to 100 µM (C–E) and 50 µm (F–K).

We generated 4x mutants from *cyp707a2-1* and *cyp707a2-2* knockout alleles by colchicine treatment (Supplemental Figure S6). Seeds generated from crosses of 2x wt with 4x wt pollen donors produced up to 65% collapsed seeds and 35% noncollapsed seeds, 27% of which could germinate (Figure 4, A–C). By contrast, crosses of both alleles of 2x *cyp707a2* with 4x *cyp707a2* pollen donors produced significantly increased numbers of noncollapsed seeds (∼60%) that were largely able to germinate (56% germinating seeds; Figure 4, A, B, D, and E). We tested whether the increased survival of 3x *cyp707a2* seeds was associated with endosperm cellularization. All 16 3x *cyp707a2* seeds analyzed had cellularized endosperm, while only two out of 22 3x wild-type seeds were cellularized (*P* = 6.88e − 09, Fischer's exact test; Figure 4, F–K). Thus, the mutation of *CYP707A2* restored endosperm cellularization and suppressed 3x seed abortion, revealing that suppressing ABA catabolism can promote 3x seed survival.

### Impaired ABA biosynthesis and signaling aggravates the triploid block

Since suppressing ABA catabolism could suppress the triploid block, we addressed whether the loss of ABA biosynthesis and signaling would aggravate this block. To test this hypothesis, we generated 4x *aba3* (*aba deficient 3*) and 4x *abi5* mutants and tested the effects of their mutations on the triploid block (Supplemental Figure S7). Mutants in *ABA3* are deficient for ABA biosynthesis (Léon-Kloosterziel et al., 1996), while the mutation of *ABI5* impairs ABA signaling (Finkelstein and Lynch, 2000).

**Figure 5:**
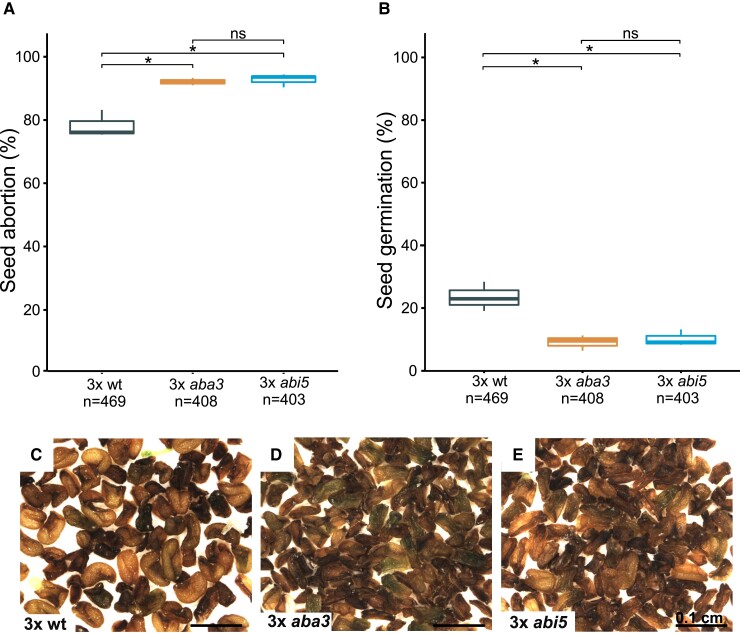
Mutations in ABA biosynthesis and signaling genes aggravate the triploid block. A, B, Phenotypic classification of 3x wt, 3x *aba3,* and 3x *abi5* seeds derived from crosses of 2x wt × 4x wt, 2x *aba3-1* × 4x *aba3-1*, and 2x *abi5-7* × 4x *abi5-7* (A), and their corresponding germination frequencies (B). Boxes show medians and the interquartile range, and error bars show the full range. C–E, Seed morphology is shown for 3x wt (C), 3x *aba3-1* (D), and 3x *abi5-7* (E). Numbers below the plots correspond to the numbers of analyzed seeds. Asterisks indicate mark significant differences. *, *P* < 0.01, relative to the control (Tukey's HSD). ns, not significant. Scale bars correspond to 100 µm (C–E).

In support of the idea that ABA can suppress the triploid block, 3x *aba3* and *abi5* seeds aborted at a significantly higher frequency than 3x wt seeds (Figure 5A) and had reduced ability to germinate (Figure 5, B–E). Together, these results reveal a critical role of ABA in 3x embryo survival and suggest that increased ABA levels confer increased desiccation tolerance to 3x embryos, thereby promoting their survival.

### ABA treatment rescues in vitro cultured triploid seeds

**Figure 6:**
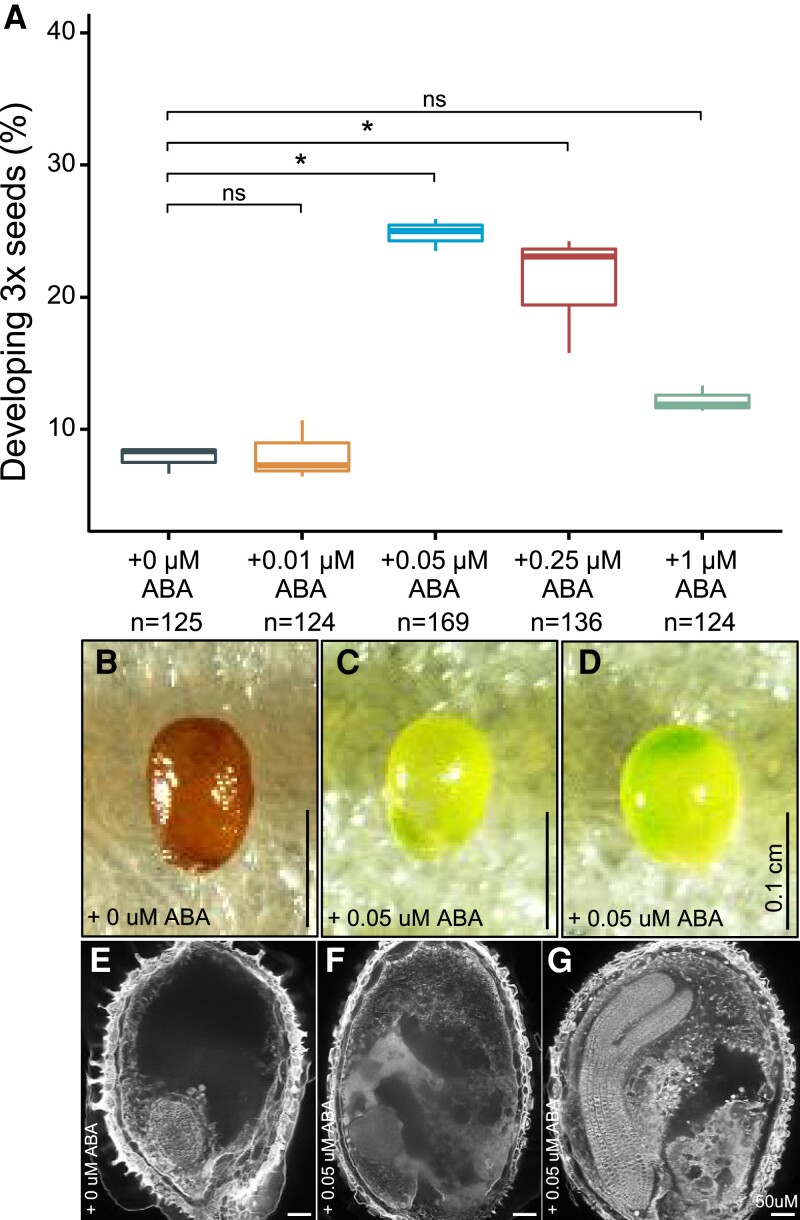
ABA can rescue 3x seeds in an in vitro culture system. A, Percentage of surviving triploid 3x seeds after 4 days of in vitro culture on medium containing 0, 0.05, 0.25, and 1 µM ABA. Boxes show medians and the interquartile range, and whiskers show the full range. B–D, Morphology of 3x seeds grown on 0.05 µM ABA (C, D) for 4 days compared to seeds grown without ABA (B). E–G, Endosperm cellularization (as determined by Feulgen staining) of seeds grown on 0.05 µM ABA (F, G) compared to seeds grown without ABA (E). *, *P* < 0.05 (Tukey's HSD). ns, not significant. Scale bars correspond to 100 µm (B–D) and 50 µm (E–G).

Exogenous application of ABA has been shown to increase stress tolerance (Fujita et al., 2005). We therefore tested whether exogenously applied ABA would increase the survival rate of 3x seeds by culturing 3x seeds containing torpedo-stage embryos on medium supplemented with ABA at different concentrations (0 µM, 0.05 µM, 0.25 µM, 0.5 µM, and 1 µM). After 4 days of in vitro culture, surviving seeds could be visually distinguished from aborting seeds, as they were plump and contained a green embryo, whereas aborting seeds were brown and started to shrivel (Figure 6, B–D). Isolated 3x wt seeds had significantly higher survival rates when cultured in vitro on medium containing up to 0.25 µM ABA compared to 3x wt seeds incubated on medium without ABA (Figure 6A), supporting the idea that ABA promotes 3x seed survival in a dose-dependent manner. After 7 days of in vitro culture, viable 3x wt embryos broke the seed coat and germinated on the medium. To determine whether ABA-mediated 3x seed survival was associated with endosperm cellularization, we analyzed endosperm cellularization in 3x seeds incubated on medium with or without ABA. All 15 green seeds incubated on ABA-containing medium had cellularized endosperm, while in the absence of ABA, only 6 out of 22 investigated seeds were cellularized (*P* = 6.07e − 06, Fischer's exact test; Figure 6, E–G); these results are consistent with the lower survival ratio of seeds grown on medium without ABA. These data support our genetic data showing increased viability of 3x seeds upon the alteration of ABA metabolism.

### Paternal-excess triploid seeds are hypersensitive to dehydration

**Figure 7:**
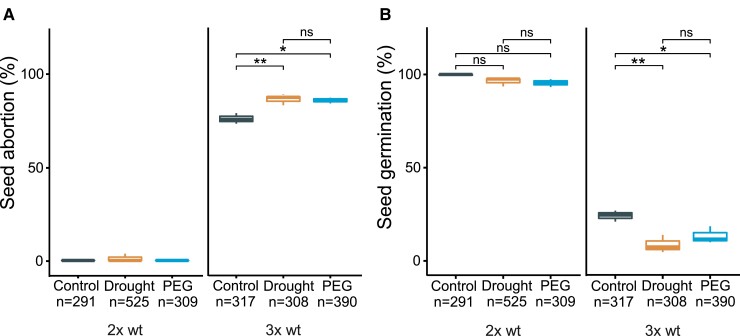
Drought enhances the triploid block. A, Phenotypic classification of 2x wt and 3x wt seeds derived from crosses of 2x wt × 2x wt and 2x wt × 4x wt after drought or PEG treatment compared to normal watering and their corresponding germination (B). Boxes show medians and the interquartile range, and whiskers show the full range. Numbers below the plots correspond to the numbers of analyzed seeds. Asterisks indicate significant differences. ns, not significant; *, *P* < 0.05; **, *P* < 0.01; relative to control (Tukey's HSD).

The increased expression of osmotic stress-responsive genes together with the rescue of triploid seeds by increasing ABA levels suggests that 3x embryos are hypersensitive to desiccation stress. To examine whether triploid block-induced osmotic stress causes seed abortion, we analyzed the viability of 3x seeds from plants grown under water limitation or treated with polyethylene glycol (PEG) to simulate dehydration (van der Weele et al., 2000; Osmolovskaya et al., 2018). Arabidopsis plants were grown under normal conditions with sufficient irrigation until flowering. After the plants were emasculated and pollinated, they were subjected to drought and PEG treatments (see Methods for details). Both treatments significantly reduced 3x seed viability, as determined by measuring seed shape and germination (Figure 7, A and B). By contrast, both treatments appeared to have only a minor effect on 2x wt seed viability, but this effect was not significant (Figure 7, A and B). The increased sensitivity of 3x seeds to dehydration stress supports the hypothesis that the triploid block induces osmotic stress to embryos that likely leads to the arrest of their development.

## Discussion

The majority of flowering plants follow the nuclear type of endosperm development, whereby initial nuclear divisions are not followed by cellularization, leading to the formation of a coenocyte (Baroux et al., 2002). In Arabidopsis, cellularization starts at the beginning of the maturation phase and proceeds in a wave-like pattern until the central cell is filled with endosperm cells (Boisnard-Lorig et al., 2001). Over a wide range of species, the failure of endosperm cellularization is connected to embryo arrest (Ramsey and Schemske, 1998; Lafon-Placette and Kohler, 2016; Köhler et al., 2021) for reasons that have remained unclear. In this study, we showed that arrested Arabidopsis embryos surrounded by an uncellularized endosperm elicit an embryo-specific osmotic stress response that resembles the seed maturation process. Seed maturation allows the seed to enter into a quiescent stage and is characterized by the acquisition of desiccation tolerance (Ali et al., 2022; Kozaki and Aoyanagi, 2022). The transcription factors ABI3 and FUS3 play central roles in regulating seed maturation in *Arabidopsis;* the loss of function of both genes results in the failure to establish desiccation tolerance and dormancy (Vicente-Carbajosa and Carbonero, 2005; Wang and Perry, 2013; Tian et al., 2020).

We found that misregulated genes in 3x embryos were enriched for sequence motifs targeted by both transcription factors, suggesting that the desiccation response was precociously activated by ABI3/FUS3 at a stage when the embryo was not yet developmentally programmed to enter the desiccation phase. This response was associated with increased ABA levels, which is similar to the increase in ABA levels that occurs during seed maturation (Holdsworth et al., 2008). ABA is a key regulator required to establish desiccation tolerance during seed maturation. Mutations in genes involved in ABA biosynthesis, sensing, and signaling impair seed dormancy (Léon-Kloosterziel et al., 1996; Lefebvre et al., 2006; Nakashima et al., 2009). Consistently, we found that mutations in ABA biosynthesis and signaling aggravated the triploid block response.

Similar to 3x embryos, Arabidopsis mutants deficient in FIS-PRC2 (FERTILIZATION-INDEPENDENT SEED-Polycomb Repressive Complex 2) components also fail to undergo endosperm cellularization and arrest their development (Ohad et al., 1996; Chaudhury et al., 1997). Embryos of *fis* mutants were shown to initiate the seed maturation program, although less robustly compared to wild-type embryos (O'Neill et al., 2019). This is consistent with our hypothesis that failed endosperm cellularization does not prevent the initiation of seed maturation, but rather it causes this program to act prematurely, leading to precocious embryo arrest. Moreover, a previous study proposed a role for ABA in promoting endosperm cellularization (Cheng et al., 2014). Consistently, we found that impaired ABA catabolism restored endosperm cellularization and thus promoted embryo survival.

Cool temperatures during early seed maturation lead to ABA retention in the endosperm upon desiccation, likely by affecting ABA catabolism during the early to mid-seed maturation period (Chen et al., 2021). Interestingly, cold treatment during seed development suppressed the abortion of interspecies hybrid seeds of *Arabidopsis thaliana* and *A. arenosa* (Bjerkan et al., 2020), which, similar to interploidy hybrids in Arabidopsis, fail to undergo endosperm cellularization (Lafon-Placette et al., 2017). It thus seems possible that the cold-induced survival of hybrid seeds is a consequence of increased ABA levels, a notion consistent with the observations made in this study.

The reason that embryos prematurely activate a desiccation response remains to be explored. We speculate that the embryo fails to properly seal the cuticle, a cutin-containing structure that builds a barrier against water loss and pathogen attack and hinders the exchange of molecules such as ABA (Moussu et al., 2013; De Giorgi et al., 2021). The cuticle is laid down during the globular stage of Arabidopsis embryo development (Szcuka and Szczuka, 2003) but is restructured during embryo development and germination (De Giorgi et al., 2021). The sealing of the embryonic cuticle requires a molecular dialogue between the embryo and the endosperm, which may require the endosperm to cellularize (Doll et al., 2020; De Giorgi et al., 2021). Nevertheless, monitoring differences in cuticle sealing in the embryo is technically challenging; therefore, testing this hypothesis remains a task of future investigations.

Together, our findings generate important insights into the requirement for endosperm cellularization and the role of ABA in this process. We also showed that endogenous and exogenously applied ABA suppressed the triploid block, opening exciting avenues for hybrid breeding.

## Materials and methods

### Plant material and growth conditions

The *Arabidopsis thaliana* mutants used in this study were described previously: *aba3-1* (Léon-Kloosterziel et al., 1996) and *nrpd1-3* (Onodera et al., 2005). The *cyp707a2-1* and *cyp707a2-2* mutants were kindly provided by Eiji Nambara and described previously (Kushiro et al., 2004). The *abi5-7* mutant (Nambara et al., 2002) was kindly provided by Ruth Finkelstein. The *osd1-1* mutant (d'Erfurth et al., 2009) was kindly provided by Raphael Mercier. As this mutant was originally identified in the Nossen background, the mutant was introgressed into the Columbia (Col-0) background by repeated backcrosses over five generations. Tetraploid Col-0 and *nrpd1-3* plants were generated using colchicine treatment as previously described (Lafon-Placette et al., 2017). Arabidopsis seeds were surface sterilized with chlorine gas (50 mL commercial sodium hypochlorite, 3 mL 25% HCl, producing ∼4% Cl_2_) for 15–30 min and plated on half-strength Murashige and Skoog (MS) medium containing 1% sucrose. After stratification for 3 days at 4°C, the plants were grown in a growth room under a long-day photoperiod (16 h light and 8 h dark, 110 μmol s^−1^ m^−2^, Valoya LED light, BX-series, NS1 spectrum) at 22°C. Eight-day-old seedlings were transferred to soil, and the plants were grown in a growth room at 60% relative humidity and daily cycles of 16 h light at 22°C and 8 h dark at 18°C. For crosses, designated female flower buds were emasculated, and the pistils were hand-pollinated at 2 days after emasculation.

### Transcriptome analysis

Embryos were dissected in 0.3 M sorbitol and 5 mM MES (pH 5.7) on a slide under a dissecting microscope at 6 DAP for diploids and 8 DAP for triploids, which in both cases corresponded to the torpedo stage of embryogenesis. The embryos were washed three times to remove contaminating endosperm. Approximately 150 embryos per sample from 3–6 independent siliques per cross were dissected in three biological replicates (crosses were made using distinct plants), and total RNA was extracted from the samples using a MagJET Plant RNA purification kit (Thermo Fisher Scientific). Messenger RNA was purified using the NEB Next poly(A) mRNA magnetic isolation module. Libraries were prepared using a TruSeq RNA Library Prep Kit v2 (Illumina) and sequenced at the SciLife Laboratory (Uppsala, Sweden) on an Illumina HiSeq2000 platform in paired-end mode.

For each replicate, 125-bp-long reads were quality trimmed using Trimmomatic (Bolger et al., 2014) and mapped to the Arabidopsis (TAIR10) genome masked for rRNA genes in pair-end mode using TopHat2 (parameters adjusted as -g 1 -a 10 -i 40 -I 5000 -F 0 -r 130; Trapnell et al., 2009). Transcript counts were calculated using GFOLD (Feng et al., 2012). Multivariate analysis (Detrended Correspondence Analysis) and Pearson's correlation test between samples were performed in order to assess the replicability and degree of similarity (Supplemental Figure S8). Differential gene expression between conditions across the three replicates was estimated using DESeq2 (Love et al., 2014). DEGs were defined as genes having a |log_2_(fold change)| > 1 with a false discovery rate adjusted *P*-value of < 0.05. The DEGs were then selected to determine enriched GO terms using the GO resource (http://geneontology.org/).

To compare our 125-bp long reads with the dataset in Martinez et al. (2018) consisting of 50-bp single-end reads, we re-analyzed the two datasets together with common customized parameters in order to reduce potential bias due to different read lengths between the two sets. Reads in both datasets were trimmed to a length of 35-bp and mapped in single-end mode to TAIR10 using HISAT2 (Kim et al., 2015) with default parameters. Transcripts per kilobase million (TPM) were estimated using StringTie (Pertea et al., 2015) and used as normalized expression values for comparison purposes.

### ABA measurement by LC-MS

For each replicate, harvested diploid and triploid seeds at the torpedo stage (∼15 mg fresh weight) were extracted using 1 mL of extraction buffer (80% MeOH, 1% Acetic acid, 19% H_2_O) and the internal standard ^2^H_6_-ABA (2 ng of ^2^H_6_-ABA in each sample) and 1 glass bead. Samples were ground in a MixerMill for 3 min at 30 Hz and centrifuged at 14,000 RPM for 10 min. 900 µL of the supernatant was transferred to a glass tube for ABA analysis. The sample was evaporated to approximately 100 µL, and 5 µL 1M HCl was added to the ABA sample. A 4 µL aliquot was injected for ABA analysis using a 6400 Series Triple Quadrupole LC-MS system.

### ABA measurement using ABAleon2.1

The plasmid containing pro*UBQ10:ABAleon2.1* (Waadt et al., 2014) Addgene (#106982) was transformed into diploid wild-type plants using the floral dip method as previously described (Clough and Bent, 1998). The T3 generation of transgenic plants was pollinated with pollen from 2x or 4x wild-type plants, and the resulting 2x and 3x seeds were observed at 6 and 8DAP, respectively, as described previously (Waadt et al., 2014) with minor modifications. Fluorescent signals were detected under a confocal Zeiss LSM780 inverted Axio microscope with a supersensitive GaASp detector with the following settings (in nanometers: excitation [ex] and emission [em]): mTurquoise, ex 458 and em 460–490; cpVenus173, ex 458 and em 510–550 using ZEN black software (Zeiss). The relative fluorescent intensities were measured using Fiji software (Schindelin et al., 2012).

### Feulgen staining

Whole siliques were fixed in ethanol:acetic acid (3:1) overnight. The samples were washed three times in water for 15 min each time, followed by 1-h incubation in freshly prepared 5 N HCl and three washes in water for 15 min each time. Staining was performed for 3 h in Schiff reagent, followed by three washes in cold water and a series of 10-min washes in a series of ethanol dilutions (10%, 30%, 50%, 70%, 90%). The samples were then incubated in 100% ethanol overnight. Embedding of the seeds was performed in a dilution series of ethanol:LR White resin (3:1, 1:1, 1:3) for 1 h each. The samples were then incubated overnight in LR White resin, mounted in LR White plus accelerator, and baked overnight at 60°C for polymerization. The seeds were imaged by two-photon confocal microscopy with excitation at 800 nm and emission >515 nm. The images were treated using Fiji software.

### In vitro seed culture

Triploid Col-0 seeds were collected at 8–9 DAP at the torpedo stage. The medium for in vitro ovule culture contained MS salt mixture (Duchefa M0222), 0.8% (w/v) plant agar, 3% (w/v) sucrose, and 0.05% (w/v) MES-KOH (pH 5.8). Immature siliques from crosses were surface sterilized for 30 s in 70% ethanol, 30 s in sterilizing solution (5% sodium hypochloride + 0.01% (v/v) of Triton X-100), and 30 s in sterile water under a sterile hood. The immature siliques were then placed on in vitro culture medium supplemented with 0, 0.01, 0.05, 0.25, and 1 µM ABA (Sigma-Aldrich) overlaid with filter paper. Each silique was opened with dissecting needles under a dissecting microscope, and all seeds were transferred onto the wet filter paper. The plates were sealed with Millipore tape and incubated under a long-day photoperiod (16 h light and 8 h dark) at 22°C.

### Drought treatment

Pots with soil were weighed before sowing to ensure equal amounts of water in the soil at the beginning of the experiment. Three independent plants were used for emasculation and pollination for each treatment. The controls were watered to keep the soil moisture at 90% until all seeds were mature and ready to harvest. For drought treatment, plants were grown under normal conditions with 90% soil moisture until pollination, after which the soil moisture content was allowed to drop to 35%. The soil moisture content was then maintained by daily watering until all seeds were mature and ready to harvest. For PEG treatment, plants were grown under normal conditions with 90% soil moisture until pollination, and were watered with 20% (w/v) PEG 8000; the PEG solution was changed daily to ensure that the PEG concentration remained unchanged until all seeds were mature and ready to harvest.

For all conditions, the pots were arranged according to a randomized design and their positions were changed daily. Three biological replicates (independent crosses from distinct plants) were collected for each sample, and drought and PEG experiments were repeated three times.

### Statistical analysis

Pairwise Fisher's exact test was performed using R version 4.2.0 and the R-package “RVAideMemoire.” One-way analysis of variance (ANOVA) followed by a post-hoc Tukey HSD test was performed using the R-package “multicomp.” Hypergeometric testing was performed using the R package “phyper.” Fisher's exact test was performed with R in a two-sided test mx = matrix. Statistical comparisons of two groups were performed by Student's *t* test in Excel. The tissue enrichment test was performed in R as previously described (Schon and Nodine 2017). All results from statistical tests are shown in Supplemental Data Set 3.

### Accession numbers

Sequence data for the genes described in this study can be found in the TAIR database (https://www.arabidopsis.org) and NCBI under the following accession numbers: *NRPD1* (AT1G63020), *OSD1* (AT3G57860), *CYP707A2* (AT2G29090), *ABA3* (AT1G16540), *ABI5* (AT2G36270). The sequencing data generated in this study are available in the Gene Expression Omnibus under accession number GSE196667.

## Supplemental Data

The following materials are available in the online version of this article.

**Supplemental Figure S1** Phenotypes of seeds derived from the indicated crosses.

**Supplemental Figure S2** Detection of RNA contamination in embryo-specific transcriptomes.

**Supplemental Figure S3** Overlap of dehydration stress- and seed maturation-specific responsive genes with differentially expressed genes (DEGs) in 3x wt embryos versus 2x wt embryos.

**Supplemental Figure S4** Log2 fold changes of gene expression levels mapped onto the KEGG pathway module.

**Supplemental Figure S5** Measurement of ABA in 2x and 3x seeds by the ABA reporter ABAleon2.1.

**Supplemental Figure S6** CYP707A2 function in ABA catabolism and generation of *cyp707a2* tetraploid mutants.

**Supplemental Figure S7** Ploidy analysis of nuclear DNA content by flow cytometry for *aba3* and *abi5* mutants.

**Supplemental Figure S8** Comparison of RNA seq samples.

**Supplemental Table S1** Quality of embryo mRNA libraries.

**Supplemental Table S2** Differentially expressed genes in 2x *nrpd1* versus 2x wt embryos.

**Supplemental Data Set 1** Fold changes of genes in embryos derived from the indicated crosses.

**Supplemental Data Set 2** Complete list of GO terms.

**Supplemental Data Set 3** Summary of statistical analyses.

## Supplementary Material

koac337_Supplementary_Data
