# Supplementary material for: Endosperm cellularization failure induces a dehydration-stress response leading to embryo arrest
Source: Plant Cell. 2022 Nov 25;35(2):874–88. doi: 10.1093/plcell/koac337 (PMC9940880; doi:10.1093/plcell/koac337)
Supplement: koac337_Supplementary_Data [file koac337_supplementary_data.zip › tpc.22.00499_Supplemental Figures and Tables.pdf]

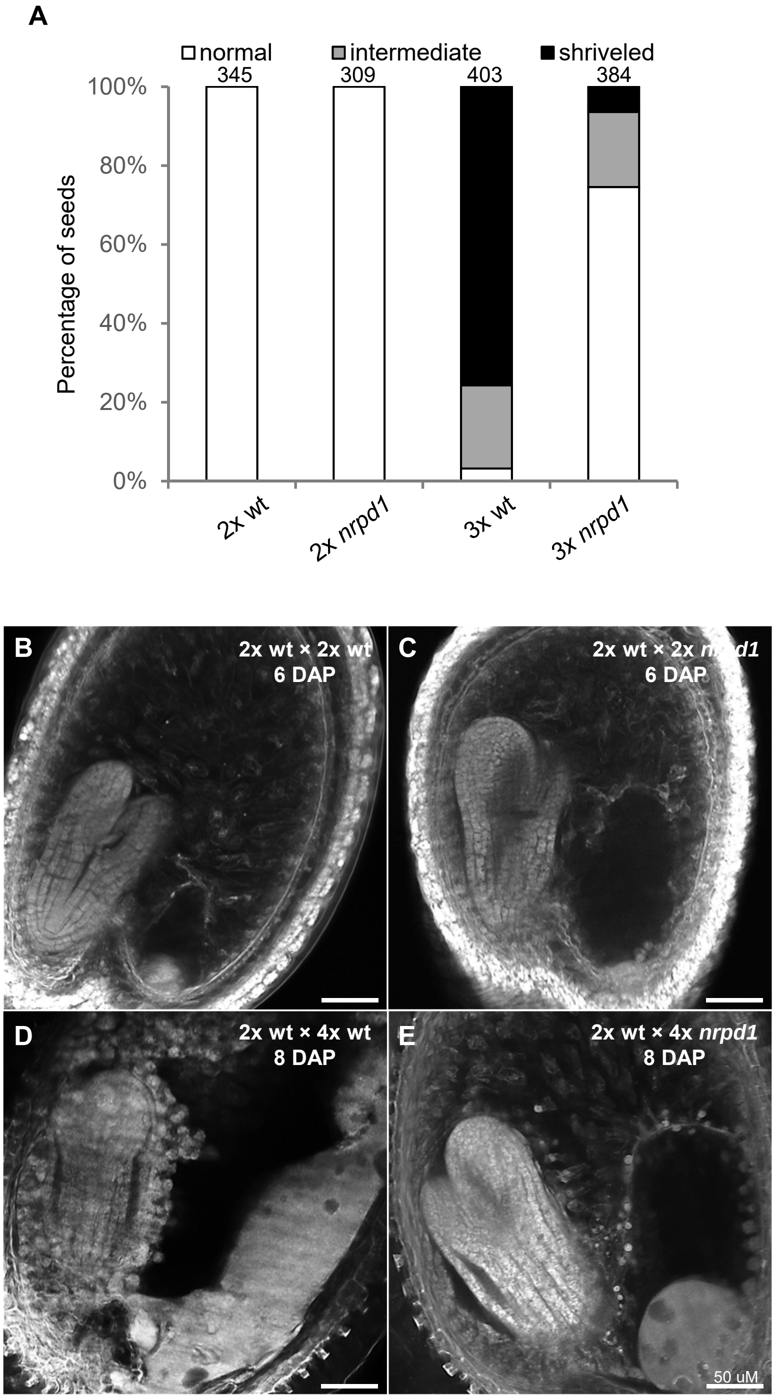

Supplemental Figure S1. Phenotypes of seeds derived from the indicated crosses. (Supports Figure 1).
(A) Phenotypic classification of seeds derived from crosses 2x wt × 2x wt (2x wt), 2x wt × 2x *nrpd1* (2x *nrpd1*), 2x wt × 4x wt (3x wt), 2x wt × 4x *nrpd1* (3x *nrpd1*). (B-E) Endosperm cellularization and embryo development as determined by Feulgen staining at 6 DAP for 2x wt seeds (B) and 2x *nrpd1* seeds (C), 8 DAP for 3x wt seeds (D) and 3x *nrpd1* seeds (E). Scale bars correspond to 50µm (B-E).

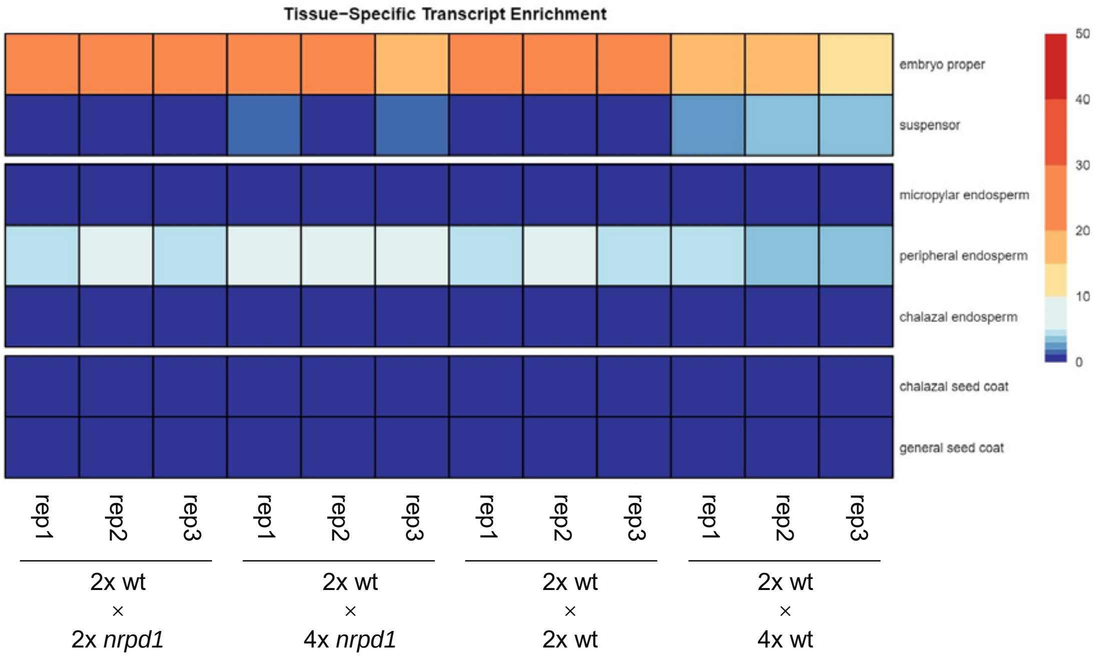

Supplemental Figure S2. Detection of RNA contamination in embryo-specific transcriptomes. (Supports Figure 1).
Heat map of tissue enrichment test results of 12 transcriptomes from embryos generated in this study.

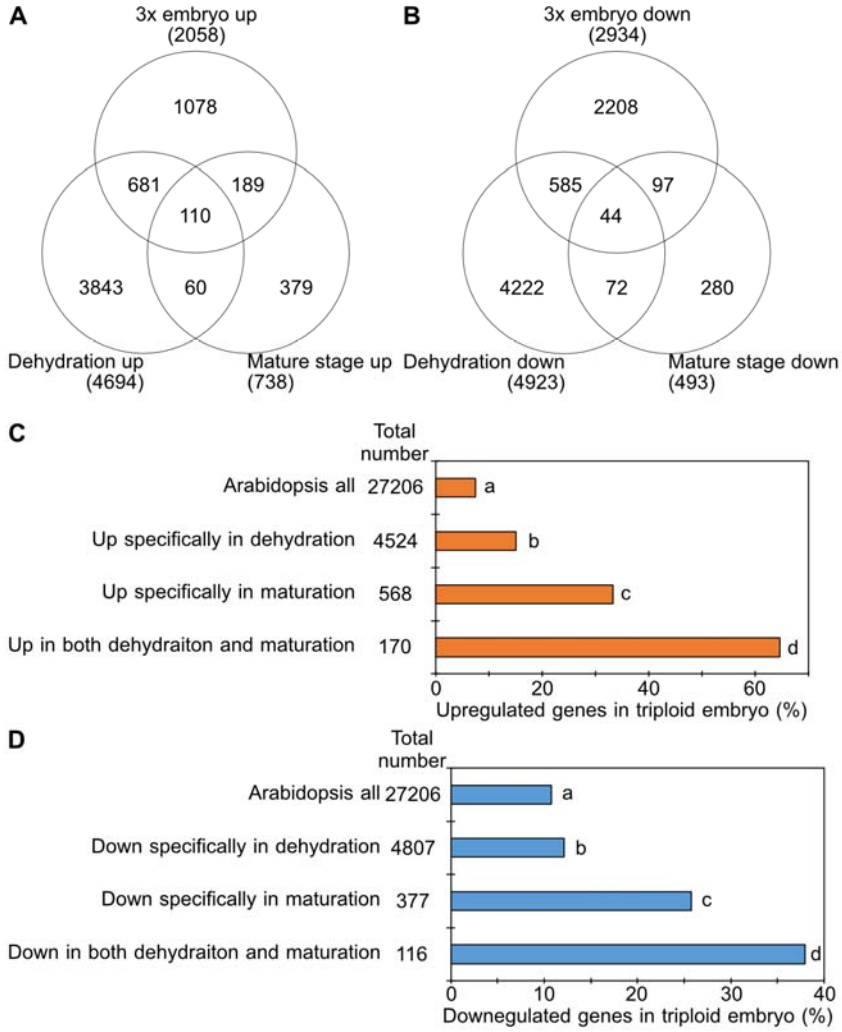

Supplemental Figure S3. Overlap of dehydration stress- and seed maturation-specific responsive genes with differentially expressed genes (DEGs) in 3x wt embryos versus 2x wt embryos. (Supports Figure 2).
(A, B) Venn diagrams showing overlapping upregulated (A) and downregulated (B) genes in dehydration stress responses (Sato et al., 2014), seed maturation (Belmonte et al., 2013) and 3x wt embryos relative to 2x wt embryos. (C, D) Percentage of DEGs in 3x embryos versus 2x embryos overlapping with upregulated (C) and downregulated (D) genes in dehydration stress response and seed maturation. Different letters beside the bars indicate significant differences (p<0.05, pairwise Fisher's exact test).

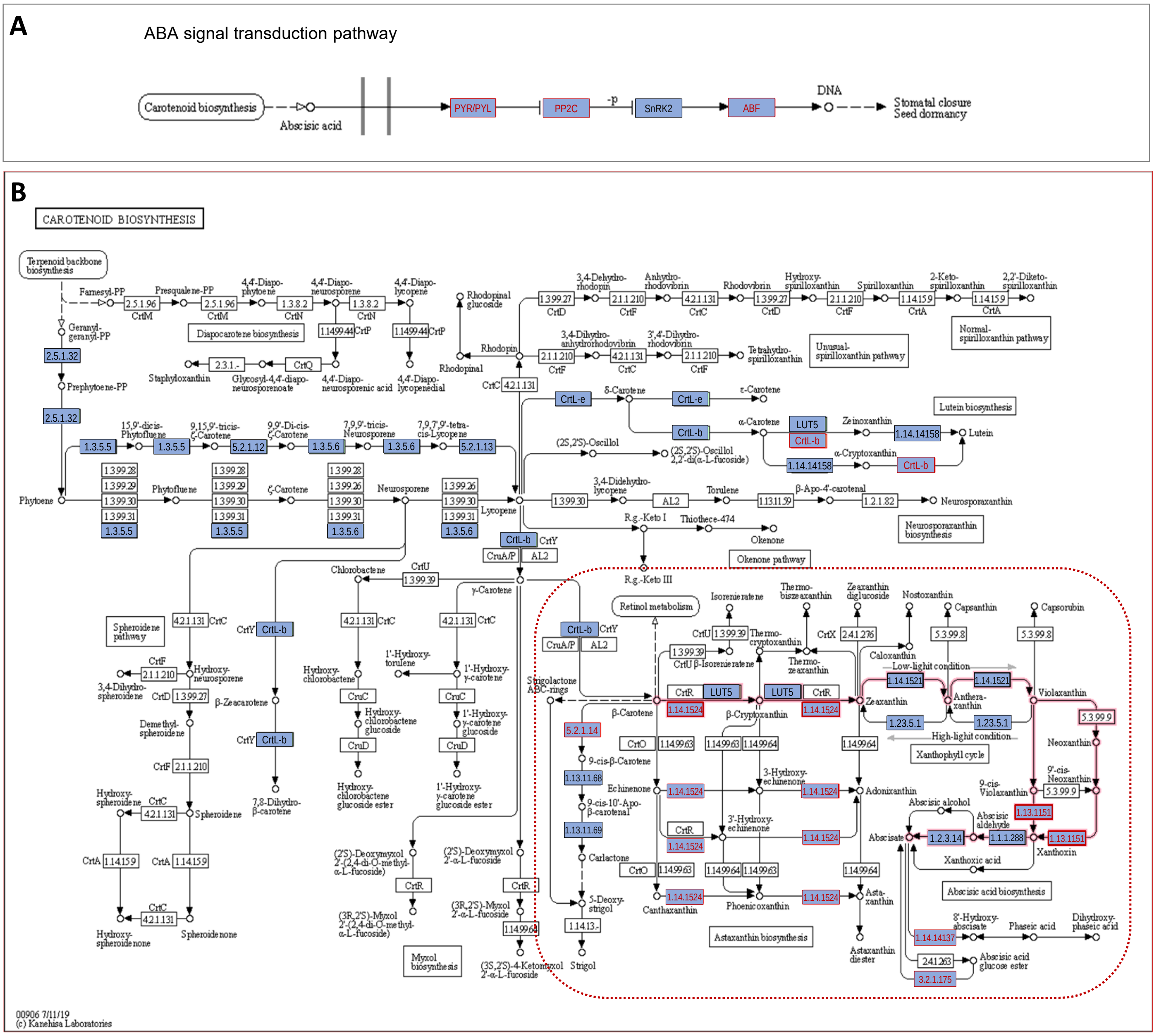

Supplemental Figure S4. Log2 fold changes of gene expression levels mapped onto the KEGG pathway module. (Supports Figure 3).

KEGG pathway of the abscisic acid (ABA) signal transduction pathway (A) and carotenoid biosynthesis pathway (B). The dotted red box marks the ABA biosynthesis pathway. Each node represents key enzymes or transporters in the pathway. Blue nodes indicate the pathway in *Arabidopsis thaliana*. Highlighted pathway module indicates the core abscisic acid biosynthesis (M00372) pathway. Nodes marked in red are associated with significantly up-regulated genes, nodes marked in black denote genes with no significant change based on the comparison 3x versus 2x wild type.

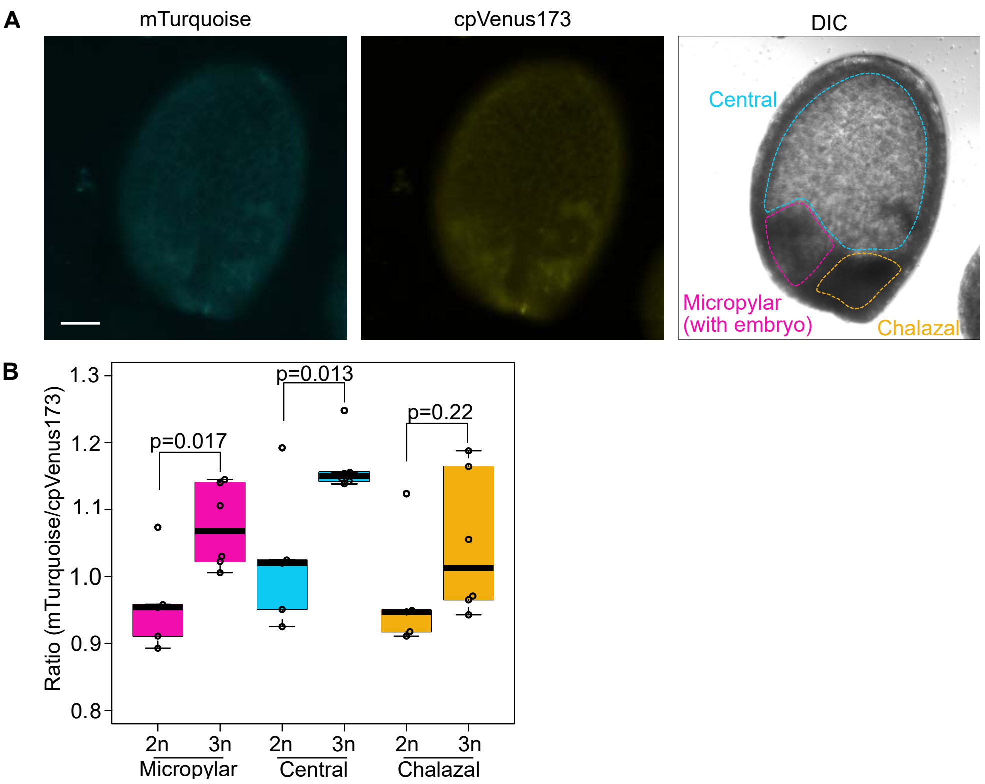

Supplemental Figure S5. Measurement of ABA in 2x and 3x seeds by the ABA reporter ABAleon2.1. (Supports Figure 3).

(A) Confocal images of mTurquoise, cpVenus174 and differential interference contrast (DIC) image of the seed expressing ABAleon2.1. The three analyzed regions are indicated in the DIC image. Scale bar corresponds to 100 µm. (B) Normalized emission ratios analyzed in three regions in 2x and 3x seeds generated by crossing between the maternal pro*UBQ10*:*ABAleon2.1* plants and paternal diploid or tetraploid wild-type plants. Boxes show medians and the interquartile range, and error bars show the full range. P values between 2x and 3x seeds in each region are shown (Student's t test, n=5-6 seeds).

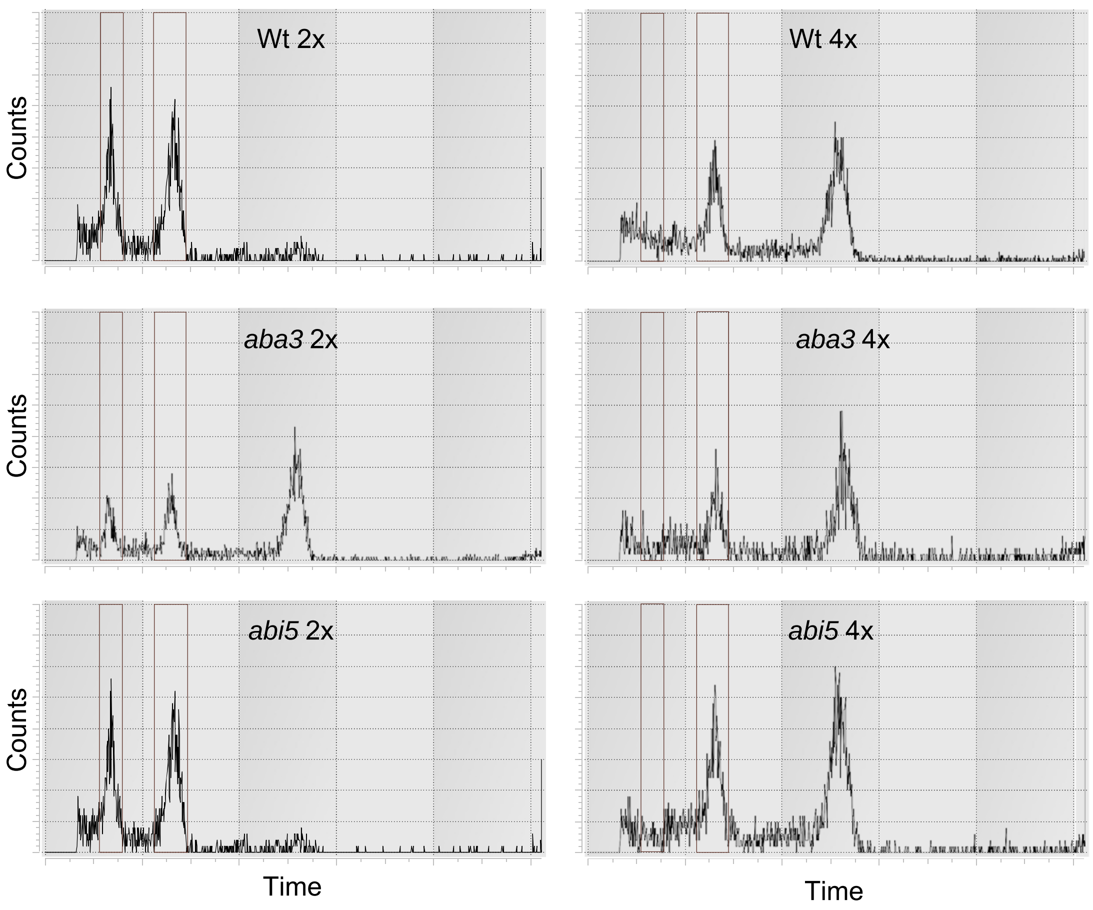

Supplemental Figure S7. Ploidy analysis of nuclear DNA content by flow cytometry for *aba3* and *abi5* mutants. (Supports Figure 5).
Nuclei isolated from 2x wt and 4x wt were used as reference standards.

Supplemental Table S1. Quality of embryo mRNA libraries.

Table shows details of the sequenced samples generated in this study. Replicates are biological replicates derived from independent crosses of distinct plants.

Sample name	Crosses	Tissue type	Total trimmed reads	Mapped reads	Map efficiency%
2x wt_rep1	Col 2x × Col 2x	6DAP-Embryo	55 300 234	37 557 760	67.9
2x wt_rep2	Col 2x × Col 2x	6DAP-Embryo	60 115 364	38 755 022	64.5
2x wt_rep3	Col 2x × Col 2x	6DAP-Embryo	40 457 047	28 037 483	69.3
2x *nrpd1*_rep1	Col 2x × *nrpd1a* 2x	6DAP-Embryo	53 159 027	33 203 321	62.5
2x *nrpd1*_rep2	Col 2x × *nrpd1a* 2x	6DAP-Embryo	66 173 212	40 897 248	61.8
2x *nrpd1*_rep3	Col 2x × *nrpd1a* 2x	6DAP-Embryo	40 403 071	25 233 945	62.5
3x wt_rep1	Col 2x × Col 4x	8DAP-Embryo	41 601 377	22 837 794	54.9
3x wt_rep2	Col 2x × Col 4x	8DAP-Embryo	48 855 276	26 400 052	54.0
3x wt_rep3	Col 2x × Col 4x	8DAP-Embryo	37 968 566	20 198 298	53.2
3x *nrpd1*_rep1	Col 2x × *nrpd1a* 4x	8DAP-Embryo	52 840 903	33 887 807	64.1
3x *nrpd1a*_rep2	Col 2x × *nrpd1a* 4x	8DAP-Embryo	33 389 971	21 545 988	64.5
3x *nrpd1*_rep3	Col 2x × *nrpd1a* 4x	8DAP-Embryo	34 885 530	20 876 554	59.8

Supplemental Table S2. Differentially expressed genes in 2x nrpd1 versus 2x wt embryos. Table shows the 9 differentially expressed genes in 2x nrpd1 compared to 2x wt embryos

id	wt2x baseMean	*nrpd1* 2x baseMean	FoldChange nrpd1 2x / wt2x	log_2FoldChange nrpd1 2x / wt2x	pval	padj
AT1G13600	9.56902053	38.0982913	3.98142016	1.99328313	8.5819E-06	0.02218214
AT1G71880	546.539738	249.394867	0.45631607	-1.1318946	4.3412E-12	7.2936E-08
AT2G36120	4.10863431	51.9941132	12.6548408	3.66161745	6.7908E-06	0.0207439
AT2G41640	99.3127714	380.592577	3.83226217	1.93819626	1.8376E-06	0.00771828
AT3G14210	14.9287367	49.7010578	3.3292206	1.73518447	1.6548E-05	0.03475376
AT4G04223	39.9381821	108.734818	2.72257804	1.4449734	3.4916E-07	0.00248679
AT5G03860	157.944078	77.6841515	0.49184593	-1.0237216	1.2749E-05	0.03060005
AT5G24240	12.2181165	42.2428347	3.45739334	1.78968475	2.8254E-05	0.04996758
AT5G35935	1.61410159	451.17109	279.518397	8.12679943	1.7861E-32	6.0015E-28
