# Supplementary material for: Endosperm cellularization failure induces a dehydration-stress response leading to embryo arrest
Source: Plant Cell. 2022 Nov 25;35(2):874–88. doi: 10.1093/plcell/koac337 (PMC9940880; doi:10.1093/plcell/koac337)
Supplement: koac337_Supplementary_Data [file koac337_supplementary_data.zip › Supplemental Fig. S6 updated.pptx]

## Slide 1
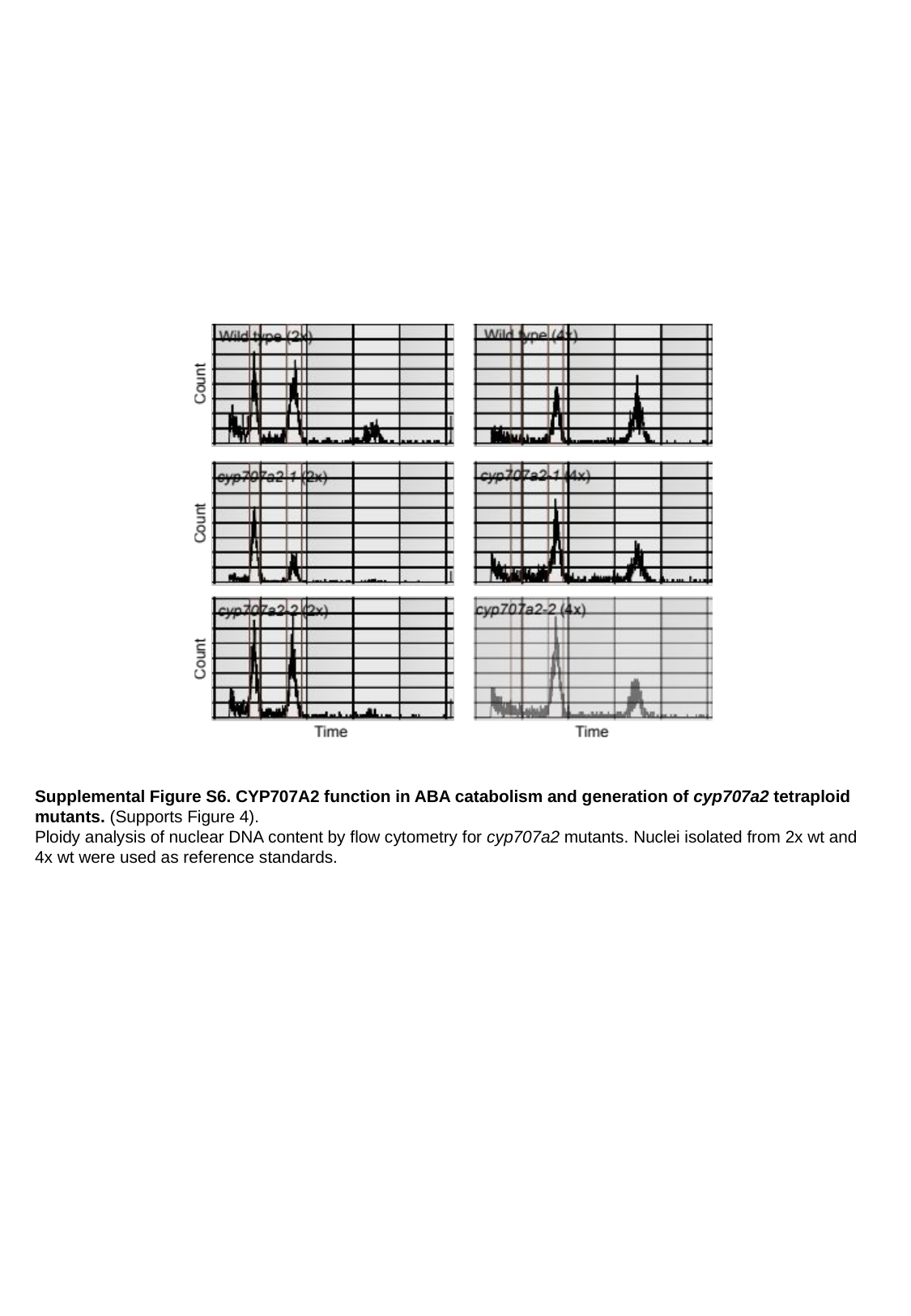

Supplemental Figure S6. CYP707A2 function in ABA catabolism and generation of cyp707a2 tetraploid
mutants. (Supports Figure 4).
Ploidy analysis of nuclear DNA content by flow cytometry for cyp707a2 mutants. Nuclei isolated from 2x wt and
4x wt were used as reference standards.
