# Supplementary material for: Endosperm cellularization failure induces a dehydration-stress response leading to embryo arrest
Source: Plant Cell. 2022 Nov 25;35(2):874–88. doi: 10.1093/plcell/koac337 (PMC9940880; doi:10.1093/plcell/koac337)
Supplement: koac337_Supplementary_Data [file koac337_supplementary_data.zip › Supplemental Fig. S8 updated.pptx]

## Slide 1
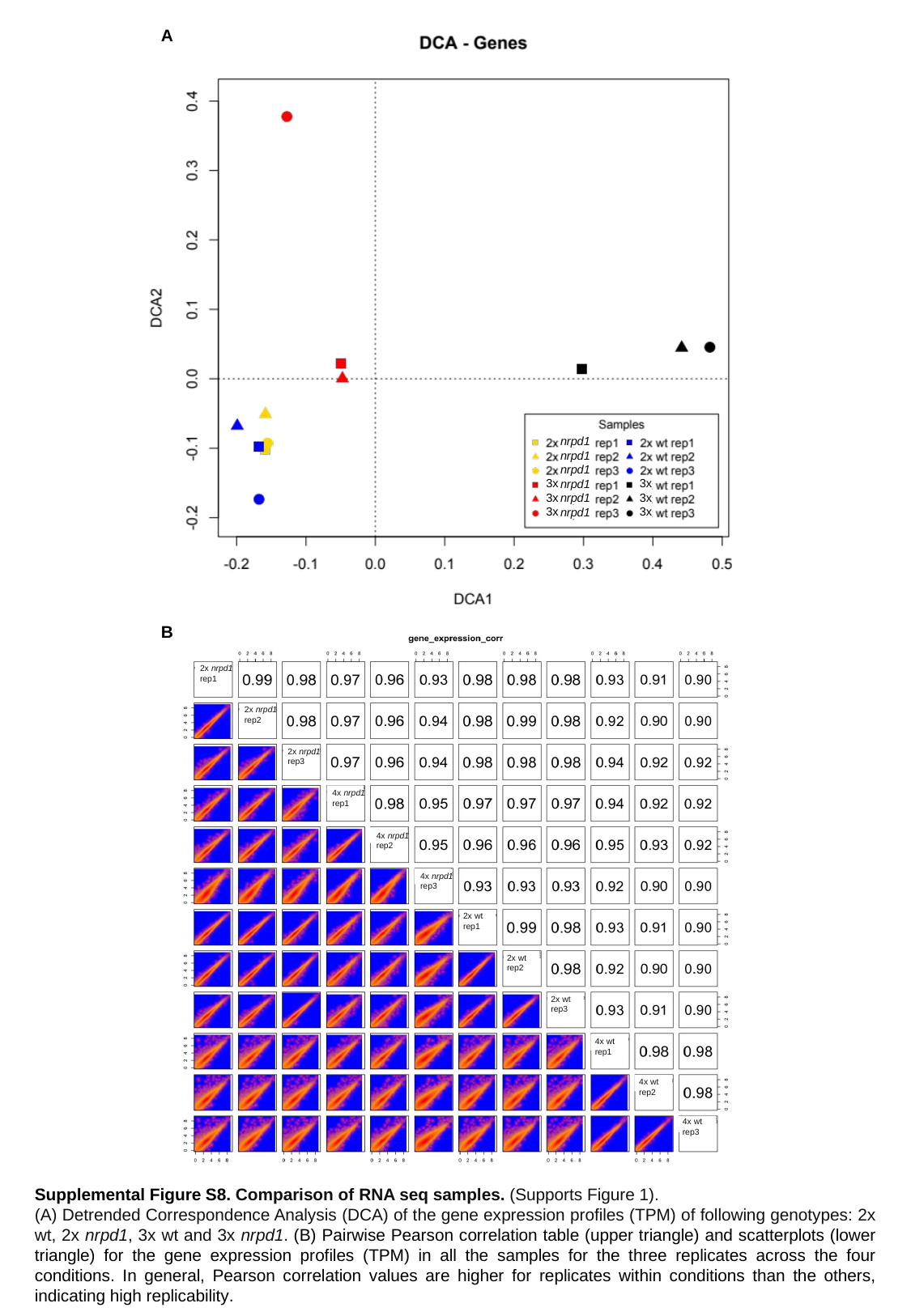

A
nrpd1
nrpd1
nrpd1
3x
3x
3x
nrpd1
3x
3x
3x
nrpd1
3x
3x
nrpd1
B
2x nrpd1
rep1
2x nrpd1
rep2
2x nrpd1
rep3
4x nrpd1
rep1
4x nrpd1
rep2
4x nrpd1
rep3
2x wt
rep1
2x wt
rep2
2x wt
rep3
4x wt
rep1
4x wt
rep2
4x wt
rep3
Supplemental Figure S8. Comparison of RNA seq samples. (Supports Figure 1).
(A) Detrended Correspondence Analysis (DCA) of the gene expression profiles (TPM) of following genotypes: 2x wt, 2x nrpd1, 3x wt and 3x nrpd1. (B) Pairwise Pearson correlation table (upper triangle) and scatterplots (lower triangle) for the gene expression profiles (TPM) in all the samples for the three replicates across the four conditions. In general, Pearson correlation values are higher for replicates within conditions than the others, indicating high replicability.
