# Supplementary material for: Endosperm cellularization failure induces a dehydration-stress response leading to embryo arrest
Source: Plant Cell. 2022 Nov 25;35(2):874–88. doi: 10.1093/plcell/koac337 (PMC9940880; doi:10.1093/plcell/koac337)
Supplement: koac337_Supplementary_Data [file tpc22.00499.PeerReviewReport.pdf › tpc22.00499.PeerReviewReport.pdf]

Endosperm cellularization failure induces a dehydration stress response leading to embryo arrest

Wenjia Xu, Hikaru Sato, Heinrich Bente, Juan Santos-González, Claudia Köhler

Corresponding author: Claudia Köhler (koehler@mpimp-golm.mpg.de)

Review timeline:

**TPC2022-RA-00499**	Submission received:	May 22, 2022
	1st Decision:	July 24, 2022 *revision requested*
**TPC2022-RA-00499R1**	1st Revision received:	October 1, 2022
	2nd Decision:	Nov 1, 2022 *acceptance pending, sent to science editor*
	Final acceptance:	Nov. 18, 2022

REPORT: (The report shows the major requests for revision and author responses. Minor comments for revision and miscellaneous correspondence are not included. The original format may not be reflected in this compilation, but the reviewer comments and author responses are not edited, except to correct minor typographical or spelling errors that could be a source of ambiguity.)

TPC2022-RA-00499 1st Editorial decision – *revision requested* **July 24, 2022**

We have received reviews of your manuscript entitled "Endosperm cellularization failure induces a dehydration stress response leading to embryo arrest." Thank you for submitting your best work to The Plant Cell. The editorial board agrees that the work you describe is substantive, falls within the scope of the journal, and may become acceptable for publication, pending revision and potential re-review.

As you will see, all reviewers are supportive, but suggest revisions that we consider reasonable. Many of these will involve text revision or re-analysis of existing data, but some new experiments are proposed. These would indeed strengthen the conclusions.

-- Reviewer comments:

Reviewer comments shown below with author responses.

TPC2022-RA-00499R1 1st Revision received **October 1, 2022**

Reviewer comments and **author responses**:

Reviewer #1:

The manuscript entitled "Endosperm cellularization failure induces a dehydration stress response leading to embryo arrest" describes the role of ABA during the triploid block of endosperm in Arabidopsis thaliana. In this study, the authors performed transcriptome analysis using the diploid and triploid embryos. The triploid embryo is aborted at the late heart stage due to lack of endosperm cellularization; however, precise molecular mechanisms are unknown. The author found that genes related to the ABA biosynthesis, transport, and transduction pathway are upregulated in triploid embryos compared to diploid endosperm. Although these findings are new information, the manuscript should be clarified the following concerns. The authors propose that the triploid embryo has increased ABA content and acquired tolerance to osmotic stress caused by uncellularized endosperm. This scenario is seemed possible since the stored hexose in the central vacuole adjacent to the embryo is converted to sucrose and consumed by the developing embryos (Hehenberger E. et al., Development 2012). The osmolarity could be increased in the uncellularized endosperm. Increased ABA levels by genetic manipulations or exogenous application restore

embryo abortion, while mutations in ABA synthesis aggravated the triploid block as embryonic arrest phenotype.

Point 1. A primary concern of this proposal is that ABA induces endosperm cellularization in addition to the proposed hypothesis, osmotic tolerance. The current experimental design could not dissect the role of ABA in cellularization and osmotic stress tolerance. Using embryo- and endosperm-specific promoters is preferable to dissect the roles of ABA; for example, endosperm-specific promoter-driven CYP707a2 in cyp702a2 mutation may address this issue.

RESPONSE: To address this concern, we performed experiments using the ABA reporter, ABAleon2.1 (Waadt et al., 2014) that allows measuring tissue-specific relative levels of ABA by calculating the emission ratios of two fluorescent proteins, mTurquoise and cpVenus173. Increased ratio of mTurquoise to cpVenus173 indicates higher accumulation of ABA. We analyzed ABA levels in three seed compartments, (1) micropylar region with embryo, (2) central and (3) chalazal regions (Supplemental Fig. 5A). We found that micropylar and central regions had significant higher levels of ABA in 3x compared to 2x seeds (Supplemental Fig. 5B), consistent with our data showing that 3x embryos developed osmotic stress responses (Fig. 1-3).

To determine whether ABA-induced 3x seed survival is associated with endosperm cellularization, we analyzed endosperm cellularization of seeds incubated on medium with and without ABA (Fig. 6). Similar to 3x *cyp707a* seeds that underwent cellularization, we also observed endosperm cellularization in seeds incubated on ABA (Figure 6E-G). We added the new data related to endosperm cellularization in triploid seeds with exogenous ABA treatment on P9, L310ff and adjusted the abstract and text (P4L106ff).

Since ABA is transported from the endosperm to the embryo by ABA transporters (Kang et al., 2015), expression of *CYP707A2* specifically in endosperm cells would not allow to eliminate the possibility that the biosynthesized ABA in the endosperm is transferred to the embryo. Conversely, expressing ABA in the embryo is likely to cause a similar leakage to the endosperm, which at least would require experimental testing. Given the problems associated with this proposed experiment together with the time requirement of nearly a year, we decided against this experiment. Nevertheless, our data show that mutants lacking ABA biosynthesis and signaling as well as drought treatment enhanced the triploid block (Figure 5 and 6), which strongly suggests that in the absence of a cellularized endosperm, ABA is likely required for 3x embryo survival.

Point 2. In addition, all experimental designs related to ABA manipulation should be checked for this issue. For example, whether exogenous application of ABA induces cellularization or not in figure 6C. ABA content should be measured using the embryos in Figure 3C, etc.

RESPONSE: To address this concern, we analyzed endosperm cellularization of seeds grown on medium with and without ABA. We found that green seeds incubated on ABA-containing medium had cellularized endosperm, while in the absence of ABA only a small fraction of seeds were cellularized ($p=6.07e-06$, Fischer's exact test) (Fig. 6E-G), consistent with the lower survival ratio of seeds grown on medium without ABA. We added these data on P9, L309ff.

Point 3. Colchicine treatment induces epigenetic change as well. Control experiments would be required. Ideally, null segregants from 4x cyp707a2 lines would be used for control. Alternatively, colchicine-treated tetraploid pollen should be used.

RESPONSE: We agree with the reviewer that colchicine treatment can induce epigenetic changes; however, over the last years working on this research topic we generated many independent colchicine induced tetraploids and never observed strong deviations from the here shown phenotype. To support this statement, we added seed morphology and germination data from F1 seeds (2x wt x 2x wt; 2x wt x 4x wt) of an additional independent colchicine induced 4x wild-type line (4x wild type-b) of that was studied in the main manuscript (4x wild type-a) (Additional Fig. 1A and 1B). As shown below, the ratios of seed abortion and germination in 3x seeds generated by using 4x wild type-b was similar to those generated by using 4x wild type-a shown in the main manuscript (Fig. 4, 5, 7 and Supplemental Fig. 1). Furthermore, we generated independent lines from two different mutant alleles *CYP707a* that both had the same phenotype (Fig 4A and 4B), making it unlikely that colchicine-induced epigenetic changes cause the observed rescue of the triploid block in 4x *cyp707a*.

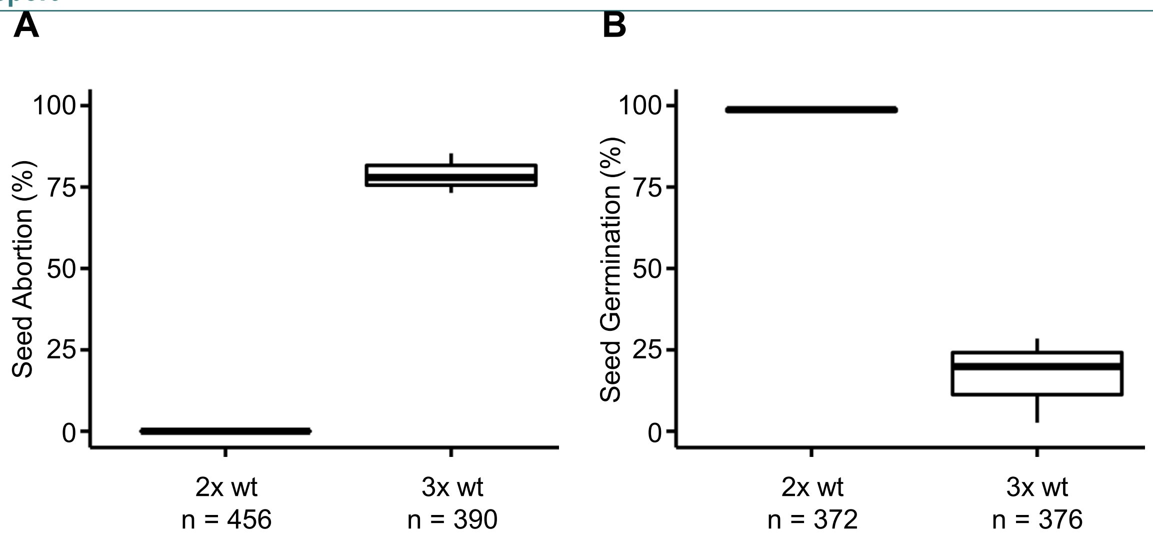

Additional Fig. 1. Phenotypes of triploid block using an additional line of the 4x wild type-b. (A) Phenotypic classification of hybrid wild type seeds in 2x wt (2x wt x 2x wt) and 3x wt (2x wt x 4x-b) and corresponding germination frequency (B) Numbers below the plot correspond to the number of analyzed seeds.

Point 4. L305 The central cell should be the central vacuole.

RESPONSE: We politely disagree, it is the central cell that includes the central vacuole that is undergoing cellularization.

<u>Reviewer #2:</u>

This manuscript describes the mechanism of failure of embryos to survive in seeds in which the endosperm aborts via paternal excess and its effect on endosperm cellularization. Transcriptome analysis suggests an enhanced response to ABA, and elevated ABA levels are confirmed in aborting embryos by LC-MS.

Strengths: The discovery that changing the ABA level can increase or decrease embryo survival will be useful for plant breeders using embryo rescue.

The key finding that ABA levels are linked to seed abortion frequency is confirmed by multiple experiments.

Point 1. Weaknesses: The transcriptome analysis is presented over three figures but in reality is a simple conclusion. So this could be condensed.

RESPONSE: To address this concern, we tried to condense the transcriptome data into one figure, but found the figure became overly large and difficult to interpret. In fact, each figure shows different analyses and different conclusions; Fig. 1 shows the genome-wide differences of the transcriptomes between 2x and 3x wild type and *nrpd1*, Fig. 2 analyzed the osmotic stress-related transcriptional profiles in 3x wt embryos and Fig. 3 confirmed the upregulation of individual ABA-related genes and higher accumulation of ABA in 3x embryos. We therefore think that the information of each figure is non-redundant and it is easiest to follow the manuscript if the data are shown in separate figures.

Point 2. The ABA levels measured are quite low. During/after cellularization ABA levels can reach about 1% of dry weight. So ABA levels are about to increase dramatically from the measured values due to maternal synthesis. A timeseries dataset would reveal how changes in ABA content are related to the timing of development.

RESPONSE: To address this concern, we performed an additional experiment to reveal the time course changes of ABA accumulation in 2x and 3x embryos (Fig. 3B). As previously shown (Okamoto et al., 2006), ABA levels increased at later developmental stages in 2x seeds. We furthermore found that triploid seeds with arrested torpedo-stage embryos and uncellularized endosperm at 10DAP had significantly higher ABA levels than 2x seeds with bent cotyledon stage embryos at 8DAP. Thus, at similar stages of embryo development, 3x seeds had significantly higher levels of ABA than 2x seeds that increased even further upon embryo arrest. We described the

data on P7, L235ff.

Point 3. Alternative models for the cause of embryo arrest have been proposed, e.g. Simonini et al., 2021. Some attempt to integrate these findings in the discussion would be welcome.

RESPONSE: Simoni et al., propose that the arrest of *mea* embryos is caused by an accelerated cell cycle. However, we found that the cell cycle-related GOs are significantly enriched among the downregulated genes (Additional Fig. 2). This suggests that the embryo growth arrest in response to the triploid block is not caused by an increased cell division activity as proposed for *mea*. The suppression of the cell cycle under osmotic stress conditions has been reported by previous studies (Lang and Schnittger, 2020);(Julkowska and Testerink, 2015), consistent with our data. However, testing these assertions would require additional experiments that we think go beyond the scope of this manuscript. We therefore prefer to not include this data into the manuscript.

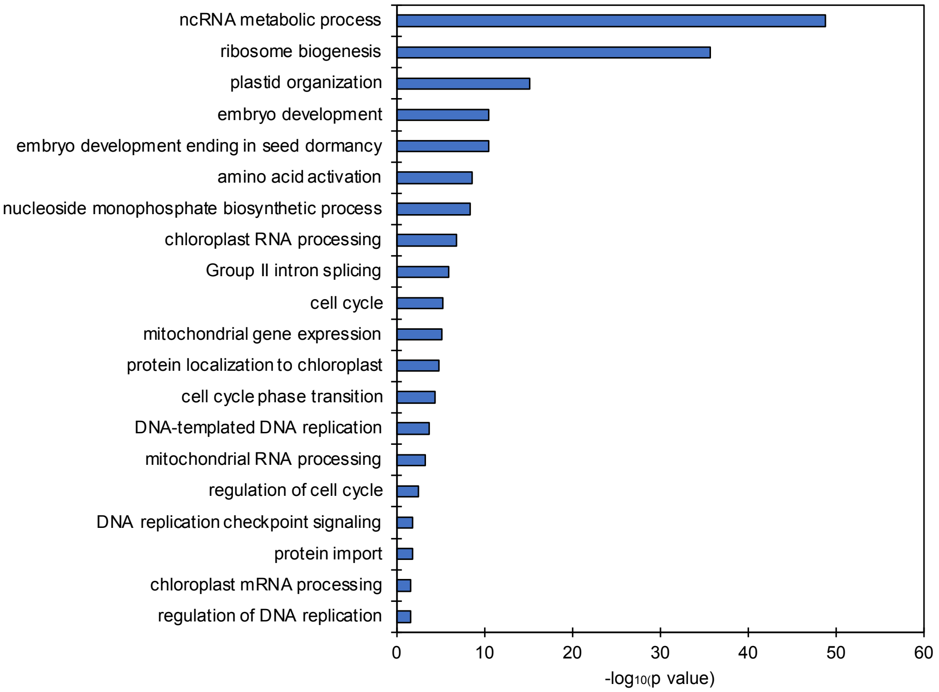

Additional Fig. 2.

Gene ontology (GO) analysis using downregulated genes in 3x wt embryos relative to 2x wt embryos. Enriched gene ontologies were analyzed using the downregulated genes in 3x wild type embryos comparing to 2x wild type embryos by PLAZA (https://bioinformatics.psb.ugent.be/plaza/).

Reviewer #3:

Xu et al. report that ABA is involved in the triploid block, leading the endosperm cellularization failure and embryo growth arrest. They performed a transcriptome analysis on torpedo-stage embryos after 2n x 2n and 2n x 4n crosses. The DEGs enriched genes related to seed maturation, especially ABA metabolism and signaling genes in upregulated genes in 3n wt. The 3n cyp707a2 mutants decreased the aborted seeds, and the 3n cyp707a2 endosperm showed cellularization. Exogenous ABA rescued in vitro culture of triploid seeds. In contrast, mutants defective in ABA biosynthesis and signaling enhanced the triploid block. Drought and PEG treatments increased seed abortion. This work includes novel and interesting findings, thus this might attract a wide range of audiences who study ploidy biology, seed development and hormone functions. The followings are my comments for the authors to be considered.

Point 1. Title and text, maturation or dehydration stress response: The authors concluded that the embryo growth arrest induces a dehydration stress response. This is based on transcriptome data, hypersensitivity of 3n embryos to stresses. However, I suggest the authors consider it more adequate to state 'seed maturation program'. First, the transcriptome data showed that ABRE and ABI3/FUS3 binding motif-like sequences were overrepresented in the

promoters. It is more maturation related process to me. Second, ABA related genes are the primary for the dehydration related DEG, but the opposite is not always true. If the authors want to stick with the dehydration response, I suggest the authors discuss DEGs to highlight the closer link to dehydration. I also suggest the authors not treat the maturation and dehydration the same. Note that CYP707A2 is induced in the maturation stage during seed development, while CYP707A1 is the predominant member around the torpedo stage in the normal 2n embryos.

RESPONSE: We agree with the reviewer and modified our manuscript accordingly, now stating that "These data suggest that the triploid block induces an embryo-specific seed maturation response that resembles a dehydration response and that is mainly regulated by B3 type transcription factors", see P5, L185ff.

We furthermore tested the similarity of dehydration-specific gene expression with the transcriptome changes in 3x embryos, by dividing the DEGs during dehydration stress (Sato et al., 2014) and seed maturation (Belmonte et al., 2013) into three groups; (1) dehydration stress-specific genes, (2) seed maturation-specific genes and (3) common genes between dehydration and seed maturation (Supplemental Fig. 3A and 3B). Every group significantly overlapped with the DEGs in 3x wt embryos relative to 2x wt embryos (Supplemental Fig. 3C and 3D), revealing that the seed maturation response mounted in 3x wt embryos has similarities with dehydration stress responses, consistent with the fact that seed maturation induces cellular dehydration (Finkelstein et al., 2002). We described and discussed the data on P5, L185ff.

Point 2. 0.3M sorbitol use for RNA-seq sampling: This is a high concentration of osmoticum, and some researchers use this condition as osmotic stress. This treatment might sensitize and enhances ABA-related DEGs in the transcriptomes. In the end, the results using the ABA-related mutants and ABA application are consistent with their model (so the model should be alright). However, this treatment may bias the interpretation of the data. Also, the osmoticum treatment is known to induce a maturation program in somatic embryogenesis. To me, overrepresentation of ABA-related DEGs is due to the hypersensitivity of the arrested embryos to the stress. The authors should describe the rationale for this treatment.

RESPONSE: The concentration of 0.3M sorbitol is commonly used for embryo dissection from seeds (Frost et al., 2018);(Gehring et al., 2006). Since we also dissected 2x embryos using the same method and gene expression in 3x embryos is compared with that in 2x embryos, the DEGs in 3x embryos should therefore be specifically associated with the triploid block rather than due to sorbitol. Furthermore, we showed the tissue-specific ABA accumulation profiles in planta in 2x wt and 3x wt seeds, and found that the micropylar region with 3x wt embryos showed a higher ABA accumulation (Supplemental Fig. 5), making it unlikely that the osmotic stress responses in 3x wt embryos relative to 2x wt embryos are a consequence of the method of the embryo dissection.

Point 3. ABA vs seed identity genes. Related to 1, I suggest the authors argue the transcriptome data in terms of seed maturation and embryo identity. The authors demonstrated the overrepresentation of B3 TF binding motifs in the DEGs. These embryo identity regulators and ABA interact with each other to form regulatory loops. Generally, the transcriptome data is discussed mainly for ABA related genes, and the authors move the argument to hydration responses. Even not too long, a more comprehensive description of transcriptome data might help convince the readers.

RESPONSE: Please see our response to the first comment which addresses this concern.

Point 4. Also, I suggest the authors describe if the overrepresentation of B3 TF motifs is associated with up-regulation of ABI3, FUS3 and LEC2 .

RESPONSE: To address this concern, we analyzed the expression levels of B3 type transcription factors, ABI3, FUS3 and LEC2 in 2x and 3x embryos in wild type and nrpd1, and added the result to Fig. 3C. The results show that the expression level of FUS3 was slightly but significantly upregulated in 3x wt embryos relative to 2x wt embryos, and the increased expression of FUS3 was suppressed in the 3x nrpd1 embryos relative 3x wt embryos (Fig. 3C). These data suggest that the overrepresentation of cis elements of B3 type transcription factors among the upregulated gene promoters in 3x wt embryos could be a consequence of increased FUS3 expression. We described and discussed the data on P7, L223ff.

Point 5. I suggest the authors to conduct reciprocal crosses to observe a similar result.

RESPONSE: The effect of maternal excess crosses (4x x 2x) is strikingly different from that of paternal excess crosses (Scott et al., 1998). While 3x seeds derived from paternal excess crosses fail to undergo endosperm cellularization (as shown here and previously), the opposite happens in maternal excess crosses where the endosperm cellularizes precociously. These distinct phenotypes are also supported by distinct transcriptome profiles ((Tiwari et al., 2010). Given the distinct phenotypes of both types of crosses, we think it would go beyond the scope of this manuscript to include the analyses of maternal excess crosses in this manuscript.

TPC2022-RA-00499R1 2nd Editorial decision – *acceptance pending* Nov. 1, 2022

We are pleased to inform you that your paper entitled "Endosperm cellularization failure induces a dehydration stress response leading to embryo arrest" has been accepted for publication in The Plant Cell, pending a final editorial review by a science editor.

Final acceptance from Science Editor Nov. 18, 2022
